# PIVOTALboost: A phase III randomised controlled trial of prostate and pelvis versus prostate alone radiotherapy with or without prostate boost (CRUK/16/018)

**DOI:** 10.1016/j.ctro.2020.08.003

**Published:** 2020-09-01

**Authors:** Isabel Syndikus, Clare Cruickshank, John Staffurth, Alison Tree, Ann Henry, Olivia Naismith, Helen Mayles, Nicola Snelson, Shama Hassan, Stephanie Brown, Nuria Porta, Clare Griffin, Emma Hall

**Affiliations:** a The Clatterbridge Cancer Centre, Wirral, UK; b Clinical Trials and Statistics Unit, The Institute of Cancer Research (ICR-CTSU), London, UK; c Cardiff University/Velindre Cancer Centre, Cardiff, UK; d The Royal Marsden NHS Foundation Trust/The Institute of Cancer Research, London, UK; e The Leeds Teaching Hospitals NHS Trust, Leeds, UK; f National Radiotherapy Trials Quality Assurance Group, The Royal Marsden NHS Foundation Trust, London, UK; g National Radiotherapy Trials Quality Assurance Group, The Clatterbridge Cancer Centre, Wirral. UK

## Abstract

• PIVOTALboost evaluates benefits/toxicity of pelvic node RT and focal boost dose escalation.
• Unfavourable intermediate/high risk and bulky local disease are most likely to benefit.
• Functional MRI imaging is used to select patients for different types of dose escalation.
• HDR brachytherapy or focal dose escalation with IMRT are used as options.
• Training and support is provided to reduce variations of contouring and radiotherapy planning.
• The trial is recruiting patients in 38 radiotherapy centres through the UK.

## 1 Introduction

Prostate cancer is the second most common cancer diagnosis in men worldwide with 1.3 million new cases in 2018 [1]. Patients with intermediate and high risk prostate cancer and those with locally advanced disease which has not spread elsewhere are recommended to have either radical prostatectomy or radical radiotherapy [2].

Four trials (CHHiP [3], PROFIT [4], HYPRO [5] and RTOG 0415 [6]) have shown moderately hypofractionated prostate radiotherapy is non-inferior to conventionally fractionated radiotherapy in terms of disease control with no consistent evidence of increased late effects. However, local, lymph node and/or biochemical failure in patients with high risk National Comprehensive Cancer Network (NCCN) disease is 20–50% [7], [8], [9], [10]. The four hypofractionation trials treated low risk (RTOG 0415), intermediate risk (CHHiP and PROFIT) and high risk (HYPRO) patients and all included the prostate and seminal vesicle as treatment volume.

The PIVOTALboost trial tests two escalation strategies in a high intermediate to high risk groups with locally bulky prostate tumours. Using functional MRI imaging, a 20 fraction schedule (moderate hypofractionation), intensity modulated radiotherapy (IMRT), and daily image guidance, it evaluates irradiating the pelvic lymph nodes and, in parallel, increasing the radiation dose to the prostate. These treatment escalation strategies need to be balanced against the risk of increased side effects which may occur if radiation dose to normal tissue is increased.

Treatment of pelvic lymph nodes using high-dose IMRT was demonstrated to be safe in the phase II PIVOTAL trial [11]. The l benefit of whole pelvic radiotherapy remains controversial; there was no long-term benefit from pelvic node treatment in the RTOG 9413 and GETUG trials [12], [13]. The outcome of RT0G 0924 (NCT01368588) and PIVOTALboost trials using modern radiotherapy techniques are therefore awaited by the clinical community [14].

Two different techniques are currently used to increase local radiation dose to the prostate with acceptable risks. High dose rate brachytherapy (HDR) delivers high doses to the whole prostate but minimises bowel and bladder irradiation [15], [16], [17]. This technique is suitable for men with significant large prostate tumour involvement and diffuse involvement. Focal dose escalation with IMRT or HDR targets intra-prostatic tumour nodules; this technique is suitable for patients with local tumour volumes <50% of the total prostate (as seen on staging MRI) [18], [19], [20]. Clinical experience indicates that this technique is feasible and safe [21], [22], [23].

## 2 Methods/study design

**Fig. 1:**
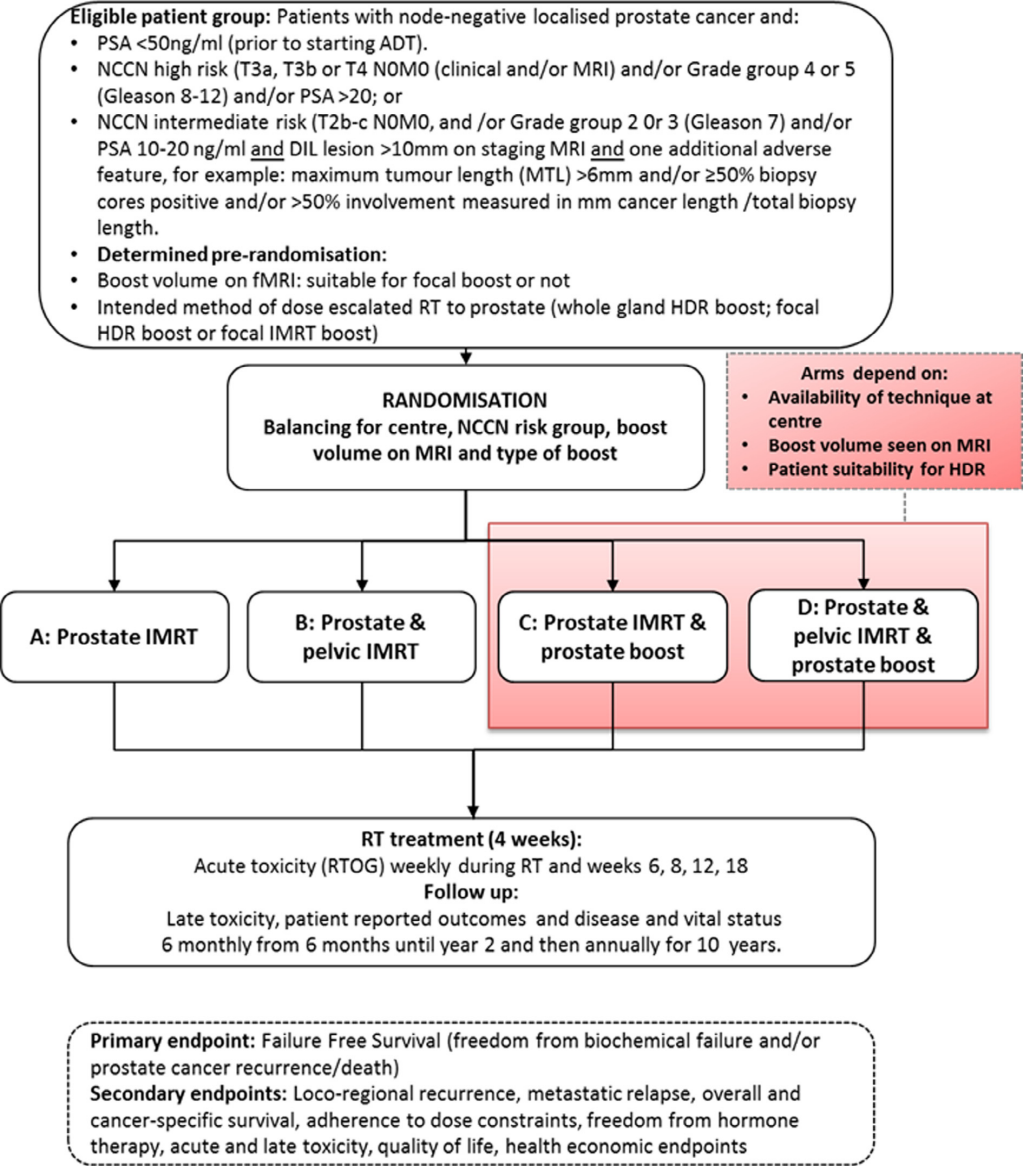
PIVOTALboost Trial Schema.

PIVOTALboost is a multicentre four-arm superiority phase III randomised controlled trial (Fig. 1; full protocol provided as appendix A). Eligible patients are allocated to one of the following treatment arms: A: prostate alone IMRT (control), B: prostate and pelvic IMRT, C: prostate IMRT and prostate boost, D: prostate and pelvic IMRT and prostate boost. All participants are considered for randomisation to arms A and B. Suitable patients with a boost volume identified by pre-biopsy MRI recruited at centres where HDR or focal IMRT is available are allocated to arms A, B, C or D.

Treatment allocation is by minimisation (with a random component) accounting for imbalances between NCCN risk groups within each stratum defined by boost-volume on MRI and type of boost.

The trial is sponsored by The Institute of Cancer Research and centrally managed by The Institute of Cancer Research Clinical Trials and Statistics Unit (ICR-CTSU). PIVOTALboost is registered (ISRCTN80146950), is part of the National Institute for Health Research Clinical Research Network Trial Portfolio and is funded by Cancer Research UK (CRUK/16/018; A20658). PIVOTALboost is supported by the National Institute for Health Research funded National Radiotherapy Trials Quality Assurance Team (RTTQA).

PIVOTALboost was approved by the UK Health Research Authority on 27th July 2017 and recruited its first patient on 4th January 2018.

### 2.1 Eligibility

Patients provide written informed consent to participate. Inclusion and exclusion criteria as follows:

#### 2.1.1 Inclusion criteria

1. Histologically confirmed, previously untreated, non-metastatic adenocarcinoma of the prostate using the Gleason scoring or grade group system.

2.1 NCCN localised high risk or locally advanced disease

• T3a, T3b or T4 N0M0 (clinical and/or MRI) and/or
• Grade group 4 or 5 (Gleason 8–10) and/or
• PSA > 20;

or

2.2 NCCN intermediate risk disease

• T2b-c N0M0, and/or Grade group 2 or 3 (Gleason 7) and /or PSA 10–20 ng/ml

and

• Dominant intra-prostatic lesion (DIL) lesion > 10 mm on staging MRI

and

• One additional adverse feature, for example: Maximum tumour length (MTL) > 6 mm and/or ≥ 50% biopsy cores positive and/or >50% involvement measured in mm cancer length /total biopsy length.

3. PSA < 50 ng/ml prior to starting androgen deprivation therapy (ADT), aged ≥18 years, written informed consent, WHO performance status 0–2.

#### 2.1.2 Exclusion criteria

1. Prior radiotherapy to prostate or pelvis, prior radical prostatectomy, adjuvant docetaxel chemotherapy.
2. Prior ADT for >6 months at consent (radiotherapy to start within 6 months of ADT start, or 12 months in case of COVID delays).
3. Radiologically suspicious or pathologically confirmed lymph node involvement, evidence of metastatic disease, life expectancy <5 years.
4. Bilateral hip prostheses, other implants/hardware making pelvic node planning difficult.
5. Contraindications to having fiducials inserted (where mandated) or undergoing a planning MRI.
6. If having HDR: long-term anticoagulation therapy which cannot be temporarily stopped, prostate surgery (TURP) with significant tissue cavity, history of recent deep vein thrombosis/pulmonary embolus, significant cardiovascular comorbidity, unfit for prolonged general anaesthetic.
7. Medical conditions making radiotherapy inadvisable.
8. Previous malignancy within the last 2 years (except basal cell carcinoma or squamous cell carcinoma of the skin), or if previous malignancy expected to significantly compromise 5 year survival.

Additional inclusion criteria for the prostate boost (arms C and D) are:

For focal boost the pre-biopsy staging multiparametric MRI (mpMRI) scan shows a dominant intra-prostatic lesion (DIL) that has:

• A score 4 or 5 lesion (clinical significant cancer is likely or highly likely to be present) according to the PI-RADS (v.2) guidelines [24]. Both T2 and DWI are important and this depends on tumour location in the gland.
• >5mm minimal axial dimension; >10 mm if patient is NCCN intermediate risk.
• Total volume estimated to be < 50% total prostate volume. If 2 or 3 DILs, total DIL volume is sum of the individual DIL volumes.

**Fig. 2:**
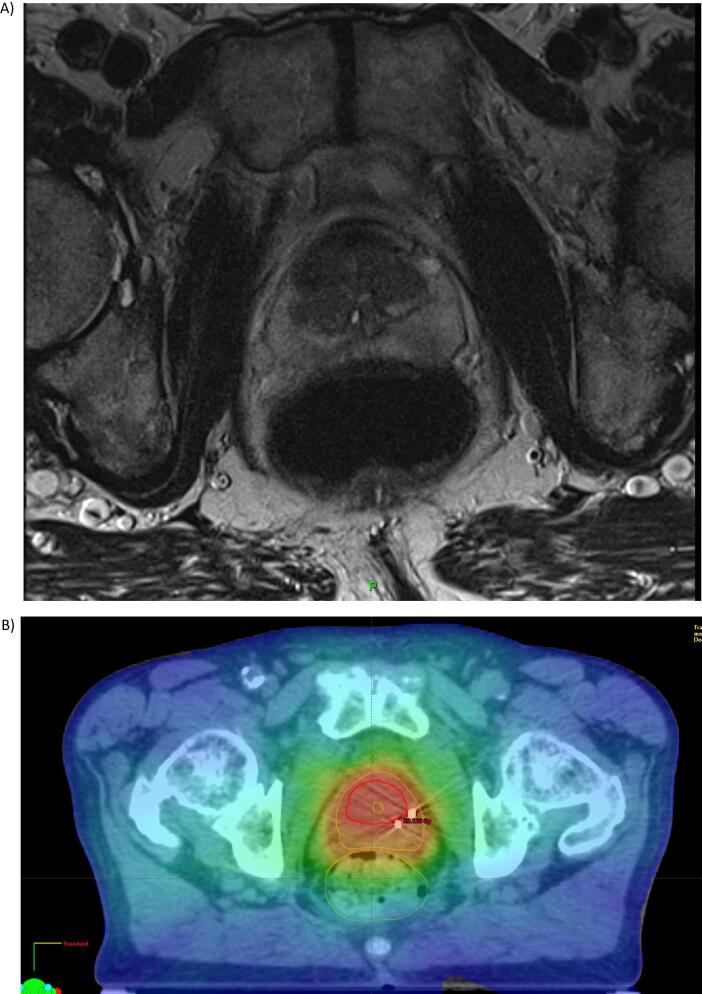
image of a suitable focal prostate boost. A) Multiparametric MRI scan at the mid gland level showing a large tumour in the central zone. B) Corresponding Planning CT scan with dose distribution around the boost volume (red contour) dose level 67 Gy (red colour)), prostate volume (orange line) dose level 60 Gy (orange colour).

Sample images of a patient suitable for focal prostate boost are given in Fig. 2.

Patients with post-biopsy MRI are not eligible for a focal boost, but can receive a whole gland boost if suitable (in the local investigator’s opinion) for HDR.

### 2.2 Study objectives

To assess whether pelvic lymph node radiotherapy with or without dose escalation to the prostate (with HDR, HDR incorporating a focal boost or focal boost IMRT) can lead to improved failure-free survival without patients experiencing increased levels of bladder (genitourinary) and bowel (gastrointestinal) side effects.

#### 2.2.1 Secondary objectives

To assess:

1) acute bladder and bowel toxicity of hypofractionated prostate +/-pelvic radiotherapy at 3 months
2) late toxicity
3) quality of life and health economics endpoints
4) time to loco-regional recurrence, time to biochemical or clinical failure, metastatic relapse-free survival, overall survival and prostate cancer specific survival, time to recommencement of androgen deprivation therapy.

#### 2.2.2 Quality of life and health economics objectives

Participants are asked to take part in a quality of life study. This includes patient reported outcomes collected using the following questionnaires: Assessment of Late Effects of RadioTherapy – Bowel screening tool (ALERT-B) [25], Gastrointestinal Symptom Rating Scale (GSRS) [26], IIEF-5 Questionnaire [27], International Prostate Symptom Score (IPSS) [28], Expanded Prostate Index Composite-26 (EPIC-26) Short Form [29].

An economic evaluation will be integrated into the design of the trial. This will be supplemented with decision modelling approaches as the benefits of intervention are likely to extend beyond the duration of the trial.

### 2.3 Trial treatment

Table 1 details the randomisation options based on the following eligibility:

• boost volume (whether the tumour volume identified on the staging MRI is suitable for focal boost or not),
• suitability and availability of HDR (e.g. patient not suitable for HDR brachytherapy or any other clinical reason) and,
• type of focal boost (IMRT or HDR brachytherapy).

**Table 1:** PIVOTALboost randomisation options.

**Randomisation Option 1 (A vs. B - Pelvic node randomisation)** No suitable focal boost volume on the staging MRI* and not suitable for HDR brachytherapy				
Arm	Radiotherapy treatment area			
	Prostate dose	Pelvic node dose		
**A**	60 Gy/20#			
**B**	60 Gy/20#	47 Gy/20#		
In centres with no access to HDR or focal IMRT boost, all patients will enter randomisation option 1 (irrespective of having a suitable boost or not).				
**Randomisation Option 2a (A vs B vs C vs D - Pelvic node and whole gland boost randomisation)** No suitable focal boost volume on the staging MRI^$^ and suitable for HDR				
Arm	Radiotherapy treatment area			
	Prostate dose	Pelvic node dose	Whole gland HDR dose	
**A**	60 Gy/20#			
**B**	60 Gy/20#	47 Gy/20#		
**C1**	37.5 Gy/15#		15 Gy/1#	
**D1**	42 Gy/20#	47 Gy/20#	15 Gy/1#	
**Randomisation Option 2b (A vs B vs C vs D - Pelvic node and focal boost randomisation)**				
Suitable focal boost volume				
Arm	Radiotherapy treatment area			
	Prostate dose	Pelvic node dose	Focal Boost dose	
			Focal IMRT**	Focal HDR**

**A**	60 Gy/20#			
**B**	60 Gy/20#	47 Gy/20#		
**C2**	60 Gy/20#		67 Gy/20#	
**C2**	37.5 Gy/15#			15 Gy/1# *(prostate)* 19 Gy/1# *(boost)*
**D2**	60 Gy/20#	47 Gy/20#	67 Gy/20#	
**D2**	42 Gy/20#	47 Gy/20#		15 Gy/1# (prostate) 19 Gy/1# (boost)

*This includes patients with post-biopsy MRI and patients with pre-biopsy MRI not fulfilling conditions for suitable boost. ^$^ this also includes patients who have a suitable boost volume at a centre where only whole gland HDR is approved.

^**^Use of focal HDR or focal boost IMRT determined for each patient prior to randomisation.

Details of the schedule of assessments and follow-up are shown in Table 2

**Table 2:** Schedule of assessments and follow up.

Visit/Assessment	Screening (pre-randomisation)	Pre-treatment	External beam treatment week 1–4	Week 6, 8, 12	Week 18	6, 12, 18, 24, 36, 48, 60 months	Annually thereafter	PSA failure or disease recurrence
Histological confirmation of prostate cancer	X							
Complete history and physical examination (physical examination & DRE if clinically indicated)	X							
WHO PS, ASA score, ACE-27 score	X							
Radiological assessment (multi-parametric MRI scan, and one of the following: bone scan, WB MRI, MRI spine, Choline PET, PSMA PET	X^1^							
PSA	X	X^4^				X	X	X
FBC, U + E	X							
Testosterone		X^4^						
Clotting and ECG		X^2^						
Baseline signs & symptoms (RTOG, CTCAE v.4)	X	X						
Acute toxicity assessment (RTOG, CTCAE v.4)		X	X	X	X			
QL questionnaires – IPSS	X		X^3^	X	X			
QL questionnaires – EPIC & EQ-5D	X				X	X		
QL questionnaires – ALERT-B, GSRS, IIEF-5 (SHIM)	X					X		
Late toxicity assessment (RTOG, CTCAE v.4)						X	X	
Assessment of disease status						X	X	X

1 Screening radiological assessment should take place ideally within 2 months and within a maximum of 12 months prior to randomisation AND no >6 months prior to starting ADT. For details how patients are screened and assessed during the COVID pandemic, please refer to section 8.1 and 8.2 of the trial protocol.

2 Only for patients randomised to HDR.

3 IPSS questionnaire to be completed only at the end of week 4.

4 At least 2 months after starting ADT and prior to starting radiotherapy. For patients who have had multiple PSAs whilst on ADT, prior to starting radiotherapy, please record the one closest to the radiotherapy start date.

### 2.4 Radiotherapy quality Assurance

A comprehensive QA programme for the PIVOTALboost trial has been designed and implemented by the National RTTQA group including pre-trial and on-trial components.

A focal boost outlining workshop was organised in June 2017. Prior to attendance at the workshop centres completed benchmark cases that RTTQA reviewed in advance of and gave feedback at the workshop. The workshop had 59 attendees from 26 sites; 21 sites submitted data for prior review. A follow-up webinar was held in September 2017.

For pre-trial QA, centres must complete the following prior to site activation: 1) Facility questionnaire, 2) Benchmark outlining cases, 3) Benchmark planning case.

On-trial QA includes: 1) Prospective and/or retrospective case reviews, 2) Dosimetry site visit (subject to prior RTTQA dosimetry accreditation) and 3) DICOM data collection for all patients.

Radiotherapy planning and delivery guidelines are provided in appendix C.

### 2.5 Safety reporting

Serious Adverse Events (SAEs) are reported after commencement of study treatment which will include fiducial marker/HDR implant insertion. In addition, RTOG grade ≥ 3 acute or late radiation side effects i.e. related to study treatment, occurring within 5 years after radiotherapy treatment are reported as SAEs.

### 2.6 Endpoints

The primary endpoint is failure-free survival defined as the time from randomisation to first biochemical failure, recommencement of androgen deprivation therapy, local recurrence, lymph node/pelvic recurrence, distant metastases or death due to prostate cancer. Secondary endpoints include time to loco-regional recurrence, time to biochemical or clinical failure, metastatic relapse-free survival, overall survival, prostate cancer specific survival, time to recommencing hormones, acute and late toxicity, quality of life and health economic outcomes.

### 2.7 Statistical considerations

PIVOTALboost is powered to detect a hazard ratio of 0.624 (equivalent to a 7% difference in 5-year failure-free survival, 87% versus 80%) for each experimental arm (B, C or D) compared to the control arm A (). For the comparison between arms A and B a total of 433 events (estimated 517 patients per group) provides 85% power (two-sided 5% significance) To achieve 80% power (with two-sided 5% significance) for the comparison between arm A and C (or D) 386 events (estimated 459 patients per group) are needed. The target sample size is therefore 1952 patients.

Treatment allocation is by minimisation using a 2:2:3:3 ratio initially as it is expected fewer sites will be able to offer boost treatment groups (C and D). Recruitment will be closely monitored and allocation ratio may be adjusted to maximise opportunity for 9:9:8:8 final relative numbers per treatment arm.

Principal analysis will occur after a median follow-up of five years or the target numbers of events have been reached. The decision to analyse at the first of these milestones will be approved by the independent data monitoring committee. Adherence to dose volume constraints will be checked after 30 patients are recruited to each experimental arm to ensure treatment can be delivered. A pre-planned interim safety analysis will be conducted after 476 participants have completed their week 18 toxicity assessment (119 per group) to rule out 30% patients with RTOG grade 2 or worse bladder or bowel complications at 18 weeks (acute toxicity) for each experimental group. There is no formal early stopping rule for futility or efficacy for the primary endpoint of failure free survival.

## 3 Discussion

The UK has a strong track record in the design and delivery of practice changing radiotherapy trials [30]. We have demonstrated that it is possible to deliver a complex radiotherapy trial supported by a comprehensive RTQA programme across a large number of UK centres, due to the ongoing enthusiasm and engagement of the UK radiotherapy community.

The primary endpoint in PIVOTALboost is failure free survival which will take 5–10 years to complete and with continued pressures on the NHS extended follow up puts a burden on the clinical and research teams. Many prostate cancer patients are discharged from secondary care after 3–5 years so the trial team will explore options for efficient collection of accurate follow-up data.

PIVOTALboost is an ambitious and potentially practice changing trial, with an efficient design addressing a number of relevant questions using modern radiotherapy techniques.

## Declaration of Competing Interest

The authors declare that they have no known competing financial interests or personal relationships that could have appeared to influence the work reported in this paper.
