# Supplementary material for: PIVOTALboost: A phase III randomised controlled trial of prostate and pelvis versus prostate alone radiotherapy with or without prostate boost (CRUK/16/018)
Source: Clin Transl Radiat Oncol. 2020 Sep 1;25:22–8. doi: 10.1016/j.ctro.2020.08.003 (PMC7508714; doi:10.1016/j.ctro.2020.08.003)
Supplement: Supplementary data 1 [file mmc1.docx]

Appendix A

PIVOTALboost protocol is at the following link:

<https://www.icr.ac.uk/our-research/centres-and-collaborations/centres-at-the-icr/clinical-trials-and-statistics-unit/clinical-trials/pivotalboost>
