# Supplementary material for: PIVOTALboost: A phase III randomised controlled trial of prostate and pelvis versus prostate alone radiotherapy with or without prostate boost (CRUK/16/018)
Source: Clin Transl Radiat Oncol. 2020 Sep 1;25:22–8. doi: 10.1016/j.ctro.2020.08.003 (PMC7508714; doi:10.1016/j.ctro.2020.08.003)
Supplement: Supplementary data 2 [file mmc2.docx]

Appendix B

**PIVOTALboost TRIAL MANAGEMENT GROUP**

Isabel Syndikus - Clatterbridge Cancer Centre

Stephanie Brown - Clinical Trials and Statistics Unit, The Institute of Cancer Research

Peter Bownes - Leeds Teaching Hospitals

Susan Campbell – Patient representative

Clare Cruickshank - Clinical Trials and Statistics Unit, The Institute of Cancer Research

Angela Flower - Clinical Trials and Statistics Unit, The Institute of Cancer Research

John Frew - The Newcastle Upon Tyne NHS Foundation Trust

Clare Griffin - Clinical Trials and Statistics Unit, The Institute of Cancer Research

Emma Hall - Clinical Trials and Statistics Unit, The Institute of Cancer Research

Shama Hassan - Clinical Trials and Statistics Unit, The Institute of Cancer Research

Vicki Hinder - Clinical Trials and Statistics Unit, The Institute of Cancer Research

Peter Hoskin - Mount Vernon Hospital

Ann Henry - Leeds Teaching Hospitals

Suneil Jain - Belfast City Hospital

Helen Mayles - Clatterbridge Cancer Centre

Olivia Naismith - Royal Marsden NHS Foundation Trust

Alfred Oliver – Patient representative

Nicola Snelson - Clatterbridge Cancer Centre

John Staffurth - Velindre NHS Trust

Chris Tierney – Patient representative

Alison Tree - Royal Marsden NHS Foundation Trust

Lucy Tregellas - Clinical Trials and Statistics Unit, The Institute of Cancer Research

Anjali Zarkar - Queen Elizabeth Hospital, Birmingham

**PIVOTALboost PROTOCOL DEVELOPMENT GROUP**

Dr Isabel Syndikus - Clatterbridge Cancer Centre

Dr Roberto Alonzi – Mount Vernon Hospital

Peter Bownes - Leeds Teaching Hospitals

Dr Brendan Carey - Leeds Teaching Hospitals

Clare Cruickshank - ICR-CTSU, The Institute of Cancer Research

Prof David Dearnaley - Royal Marsden NHS Foundation Trust

Dr John Frew - The Newcastle Upon Tyne NHS Foundation Trust

Clare Griffin - ICR-CTSU, The Institute of Cancer Research

Prof Emma Hall - ICR-CTSU, The Institute of Cancer Research

Dr Ann Henry - Leeds Teaching Hospitals

Prof Peter Hoskin - Mount Vernon Hospital

Laura Howard - Clatterbridge Cancer Centre

Dr Suneil Jain - Belfast City Hospital

Dr Vincent Khoo - Royal Marsden NHS Foundation Trust

Rebecca Lewis - ICR-CTSU, The Institute of Cancer Research

Dr Anna Lydon - Torbay and South Devon Hospital

Helen Mayles - Clatterbridge Cancer Centre

Olivia Naismith - Royal Marsden NHS Foundation Trust

Dr Ashok Nikapota - Sussex Cancer Centre

Dr Peter Ostler - Mount Vernon Hospital

Dr Heather Payne - UCL Hospital, London

Dr Nuria Porta - ICR-CTSU, The Institute of Cancer Research

Dr Yvonne Rimmer - Addenbrooke’s Hospital

Dr Azmat Sadozye - NHS Greater Glasgow and Clyde, Glasgow

Dr John Staffurth - Velindre NHS Trust

Dr Alison Tree - Royal Marsden NHS Foundation Trust -

Dr Maria Schmidt - Royal Marsden NHS Foundation Trust

Dr Christopher Scrase - Ipswich Hospital NHS Trust

Dr Chris South - Royal Surrey County Hospital, Guildford

Dr Paula Wells - St, Bartholomew’s Hospital, London

Dr Anjali Zarkar - Queen Elizabeth Hospital, Birmingham

|  |  |  |
| --- | --- | --- |
