# Supplementary material for: PIVOTALboost: A phase III randomised controlled trial of prostate and pelvis versus prostate alone radiotherapy with or without prostate boost (CRUK/16/018)
Source: Clin Transl Radiat Oncol. 2020 Sep 1;25:22–8. doi: 10.1016/j.ctro.2020.08.003 (PMC7508714; doi:10.1016/j.ctro.2020.08.003)
Supplement: Supplementary data 3 [file mmc3.pdf]

PIVOTALboost

A phase III randomised controlled trial of prostate and pelvis versus prostate alone radiotherapy with or without prostate boost

RADIOTHERAPY PLANNING AND DELIVERY GUIDELINES

Version: 2.2

Dated: 20/12/2019

Chief Investigator: Dr Isabel Syndikus

Clinical Co-ordinators: Prof John Staffurth, Dr Ann Henry, Dr Alison Tree

Sponsor: The Institute of Cancer Research

Funders: Cancer Research UK

Coordinating Trials Unit: ICR Clinical Trials and Statistics Unit (ICR-CTSU)

The Institute of Cancer Research

Main REC Reference Number: 17/LO/0731

ISRCTN: ISRCTN80146950

This is a controlled document which should be referred to in conjunction with the PIVOTALboost protocol and should not be copied, distributed or reproduced without the written permission of the PIVOTALboost Trials Office – PIVOTALBoost-icrctsu@icr.ac.uk

ADMINISTRATION

Clinical Coordination

Dr Isabel Syndikus
(Chief Investigator)
Clatterbridge Cancer Centre
Bebington, Wirral
CH63 4JY
Tel: 0151 4827685
Isabel.syndikus@nhs.net

Prof John Staffurth
Clinical co-ordinator (nodal radiotherapy) and translational studies lead
Velindre Cancer Centre
Cardiff CF14 2TL
Tel: 02920 316964
John.Staffurth@wales.nhs.uk

Dr Ann Henry
Clinical co-ordinator (High dose rate brachytherapy)
St James's University Hospital, Leeds, LS9 7TF
0113 206 7630
a.henry@leeds.ac.uk

Dr Alison Tree
Clinical co-ordinator (simultaneous integrated boost)
Royal Marsden Hospital
Sutton, SM2 5PT
020 8642 6011
alison.tree@rmh.nhs.uk

ICR-CTSU Scientific & Methodology Lead : Prof Emma Hall
Tel: 020 8722 4013
Emma.Hall@icr.ac.uk

ICR-CTSU Statistician: Clare Griffin / Vicki Hinder
Tel: 020 8722 4101
PIVOTALboost-icrctsu@icr.ac.uk

ICR-CTSU Senior Trial Manager: Clare Cruickshank
Tel: 020 8722 4058
PIVOTALboost-icrctsu@icr.ac.uk

Trial Manager: Shama Hassan/ Stephanie Brown
Tel: 020 8722 4183/4467
PIVOTALboost-icrctsu@icr.ac.uk

Any questions relating to the Radiotherapy Planning document should be addressed in the first instance to the PIVOTALboost RTQA team:

Radiotherapy Quality Assurance Team RTQA Physicists, email: pivotalboost.trial@nhs.net

Olivia Naismith
Royal Marsden Hospital, London SW3 6JJ
Tel: 020 7808 2500
Email: Olivia.Naismith@rmh.nhs.uk

Helen Mayles and Nicola Snelson
Clatterbridge Cancer Centre, Merseyside
CH63 4JY
Emails: Helen.Mayles@nhs.net
Nicola.Snelson1@nhs.net

1. INTRODUCTION AND TRIAL SUMMARY 5
1.1. TRIAL SCHEMA 7
2. TRIAL ARMS 8
2.1. The Boost Volume 8
3. PROCEDURES BEFORE PLANNING 8
3.1. Staging MRI Imaging 8
3.2. Androgen Deprivation Therapy (ADT) 9
3.3. Image Guidance (IGRT) 9
4. PLANNING CT AND MRI SCANS FOR EXTERNAL BEAM RADIOTHERAPY 9
4.1. Patient Preparation and Positioning 9
4.2. Planning Scans 10
5. ORGANS AT RISK AND TARGET VOLUME DEFINITIONS FOR EXTERNAL BEAM PLANNING 11
5.1. Organs at Risk (OAR) 11
5.2. Target Volume Definition 12
5.3. GTV and CTV Definition 12
5.4. Prostate Volumes 13
5.5. Clinical Target Volumes and Planning Target Volumes (for External Beam RT) 14
6. EXTERNAL BEAM RADIOTHERAPY PLANNING GUIDELINES 15
6.1. Radiotherapy Technique 15
6.2. Prescribed Dose and Fractionation 15
6.3. Definition of PTVs for Dose Reporting with their Dose Constraints 16
6.4. Normal Tissue Dose Constraints for Organs at Risk for External Beam Plans 17
6.5. Extra Considerations concerning Treatment Delivery 18
7. WHOLE GLAND HDR and FOCAL HDR: TARGET VOLUMES FOR ARMS C1, D1, C2 AND D2 19
7.1. Brachytherapy Reporting Parameters 20
8. RADIOTHERAPY DELIVERY 20
8.1. IGRT (please also refer to APPENDIX B. GENERAL GUIDELINES FOR IGRT) 20
8.2. Fiducial Markers and kV Imaging 21
8.3. Cross Sectional Imaging (CBCT, CT on Rails, Tomotherapy) 21
8.4. Cross Sectional Imaging (Ultrasound) 21
9. TREATMENT SCHEDULING 21
10. DOCUMENTATION ON COMPLETION OF RADIOTHERAPY 21
11. RADIOTHERAPY QUALITY ASSURANCE 21
11.1. Radiotherapy Quality Assurance Overview 21
11.2. Pre-Trial Questionnaires 22
11.3. Benchmark Cases and downloading date from RTTRIALS website 22
11.4. Outlining Benchmark Cases 23
11.5. Focal Boost Outlining Examples 24

11.6. Planning Benchmark Case ... 24
11.7. HDR Planning Benchmark ... 24
11.8. Patient Case Reviews ... 29
11.9. Dosimetry Audit ... 30
11.10. Ongoing Data Collection ... 30
11.11. DICOM Data Export ... 31
12. REFERENCES ... 32
13. APPENDIX A. (Diagnostic) MRI for PIVOTALboost ... 33
13.1. Patient Preparation ... 33
13.2. MRI sequences ... 33
13.3. Magnetic Field Strength ... 33
13.4. Endorectal Coil ... 33
13.5. Technical Specifications ... 34
14. APPENDIX B. GENERAL GUIDELINES FOR IGRT ... 36
14.1. Patients with Fiducials ... 36
14.2. Patients without Fiducials ... 37
14.3. Proposed Actions to be taken during treatment with IGRT when the difference between the prostate match and the nodes match exceeds 5 mm ... 38

Major Revisions since previous Version

Version No.	Major Revisions
Version 1.1	First Version
Version 2.0	Section 1: Amended to agree with current protocol version<br>Section 3.3: Clarification of use of fiducial markers<br>Section 6.4: Reduction of mandatory Bowel V40Gy DVH constraint to 70 cm^3<br>Section 11.3: Addition of instruction as to how to download from RTTQA website<br>Section 11.7: Addition of Instructions for planning HDR benchmark<br>Section 11.8: List of review schedules for on-trial patients<br>Section 14 Appendix B: Recommended IGRT process for a Linac with 2D Marker matching
Version 2.1	Section 3.3: Amended to agree with protocol amendment, that fiducial markers are no longer mandatory for Arms C2 and D2<br>Section 6.3: PTVp and CTVpb median dose constraints amended for IMRT boost arms C2 and D2.<br>Section 14 Appendix B: General Guidelines for IGRT now includes CBCT only guidelines.<br>Section 14.3 added to give guidelines as to how to proceed when prostate and nodes do not match within tolerance.
Version 2.2	Section 5.5, Table 5-3 - Table 5-5: Margins for PTVp and PTVpsv increased to 4mm and 8mm for patients without fiducials<br>Section 11.11: New instruction for sending DICOM data to Trial Physicists

1. INTRODUCTION AND TRIAL SUMMARY

PROTOCOL TITLE	PIVOTALboost: A phase III randomised controlled trial of prostate and pelvis versus prostate alone radiotherapy with or without prostate boost
TARGET DISEASE	1. Histologically confirmed, previously untreated, non-metastatic adenocarcinoma of the prostate<br>2. PSA < 50 ng/ml (prior to starting ADT)<br>3. NCCN localised high risk or locally advanced disease<br>• T3a, T3b or T4 N0M0 (clinical and/or MRI) and/or<br>• Grade group 4 or 5 (Gleason 8-10) and/or<br>• PSA > 20; or<br>4. NCCN intermediate risk disease<br>• T2b-c N0M0, and/or Grade group 2 or 3 (Gleason 7) and/or PSA 10-20 ng/ml<br>and<br>• DIL lesion >10mm on staging MRI<br>and<br>• One additional adverse feature, for example: maximum tumour length (MTL) >6mm and/or ≥50% biopsy cores positive and/or >50% involvement measured in mm cancer length /total biopsy length.
STUDY OBJECTIVES	The primary objective of PIVOTALboost is to assess whether pelvic lymph node radiotherapy with or without dose escalation to the prostate with HDR, HDR incorporating a focal boost, or focal boost IMRT can lead to improved failure-free survival with similar levels of bladder (genitourinary) and bowel (gastrointestinal) side effects experienced by patients.
STUDY DESIGN	Multicentre four-arm phase III randomised controlled trial
TRIAL POPULATION	Patients receiving radical radiotherapy for localised, node negative prostate cancer
RECRUITMENT TARGET	1952 patients
TRIAL TREATMENT	Patients will be allocated to one of the following treatment arms:<br>• A: Prostate alone IMRT<br>• B: Prostate and pelvic IMRT<br>• C: Prostate IMRT and prostate boost<br>• D: Prostate and pelvic IMRT and prostate boost.<br>Randomisation into arms C and D will depend on the boost volume identified by MRI (suitable for focal boost or not), availability of focal HDR or IMRT, and patient suitability in the case of HDR
NEOADJUVANT AND ADJUVANT HORMONE THERAPY	Patients in the intermediate risk group will receive 6-12 months of ADT, and patients in the high risk group may receive 2-3 years of ADT; patients ideally undergo planning after 2-4 months (maximum 6) of neo-adjuvant ADT and commence radiotherapy at a maximum of 6 months.
PRIMARY ENDPOINT	Failure-free survival (FFS), defined by the time to first biochemical failure, recommencement of androgen deprivation therapy, local recurrence,

	lymph node/pelvic recurrence, distant metastases, or death due to prostate cancer.
SECONDARY ENDPOINTS	• Time to loco-regional recurrence; time to biochemical failure or prostate recurrence; metastatic relapse free survival; overall and prostate cancer specific survival; time to recommencement of androgen deprivation therapy.<br>• Adherence to dose constraints.<br>• Acute bladder and bowel toxicity at 3 months.<br>• Late toxicity.<br>• Quality of life.<br>• Health economic endpoints.
FOLLOW UP	**RT treatment (1-18 weeks):**<br>• Acute toxicity (RTOG, CTCv4.0) assessed during radiotherapy week 1-4, week 6, 8 12 and 18, plus QL from start of RT.<br>**Follow up:** *time points from start of RT*<br>• Late toxicity and PSA at 6, 12, 18, 24 months, annually for 5 years<br>• Annual follow-up for PSA and recurrence at least until 10 years.

1.1. TRIAL SCHEMA

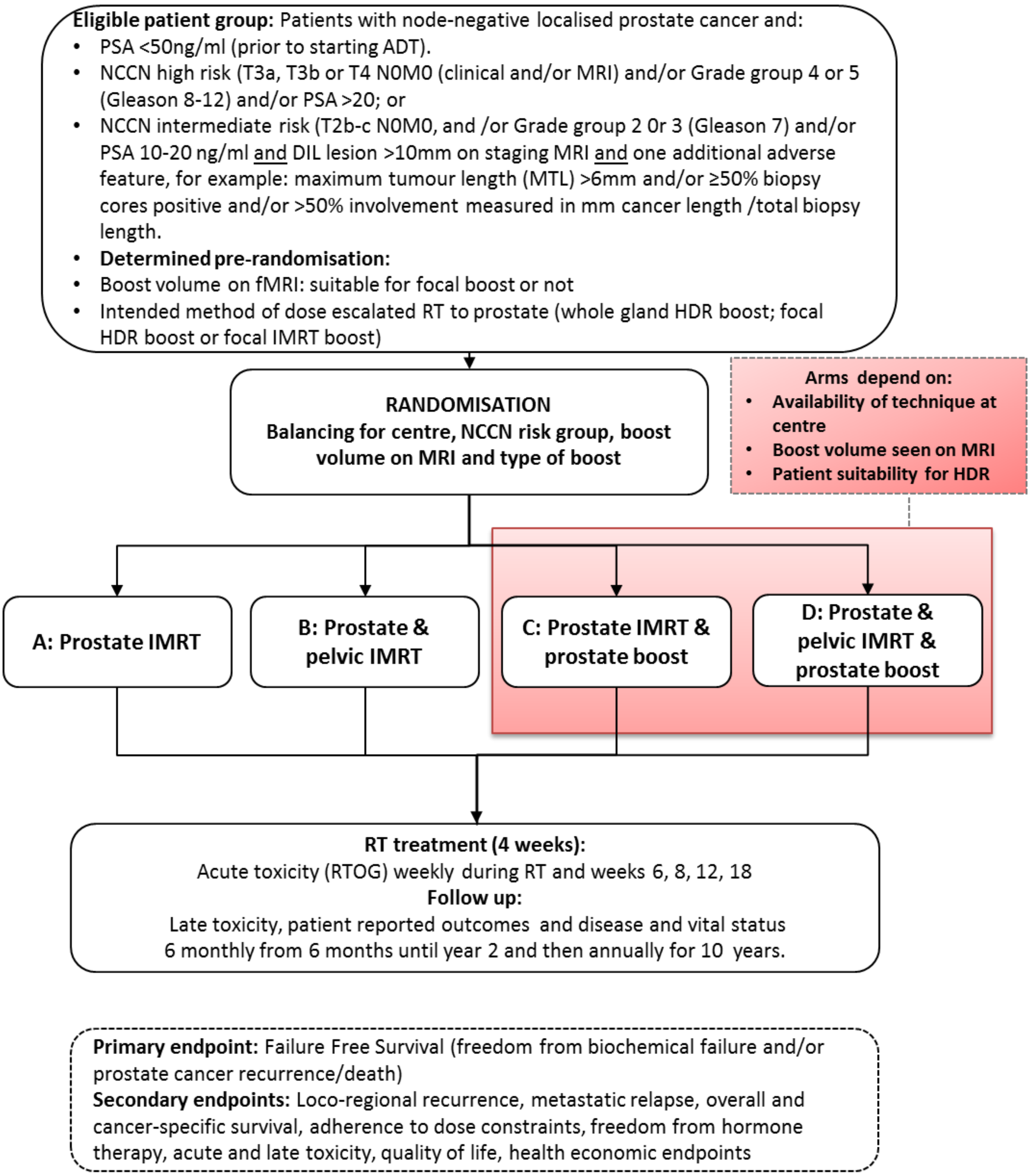

All patients will receive inverse-planned prostate IMRT delivered in 15 or 20 fractions with daily online image-guided radiotherapy (IGRT). Various IMRT delivery techniques are allowed (static field, VMAT, helical tomotherapy, robotic gantry) and all will be referred to as IMRT in this document unless specifically detailed. The delivery method for the prostate boost will vary between patients and between centres based on MRI characteristics, patient characteristics, techniques available and patient/clinician choice, as discussed in detail in the main protocol. Ideally, radiotherapy treatment should commence on Weds, Thurs, or Fri (see section 9).

2. TRIAL ARMS

Participants allocated to **arm A or B** will have:

- One radiotherapy plan generated to deliver all 20 fractions in a single phase IMRT technique with a full bladder and empty rectum; radiotherapy will be given in 5 fractions per week with daily online IGRT.

Participants allocated to **C1 or D1 (whole gland HDR)** or **C2 and D2 (focal HDR boost)** will have:

- A single fraction HDR brachytherapy implant without (C1 and D1) or with a focal boost (C2 or D2) in addition to 2-3 weeks later 15 fractions (C1 or C2) or 20 fractions (D1 or D2) of external beam radiotherapy (EBRT) delivered in a single phase IMRT technique with a full bladder and empty rectum; radiotherapy will be given 5 fractions per week with daily online IGRT, and is usually given 2-3 weeks after the implant. (It is permitted for the HDR treatment to follow the EBRT if that is the Centre's practice).

Participants allocated to **C2 and D2 (focal boost IMRT option)** will have:

- One radiotherapy plan generated to deliver all 20 fractions in a single phase IMRT technique with a full bladder and empty rectum; radiotherapy will be given in 5 fractions per week with daily online IGRT.

2.1. The Boost Volume

Patients with a suitable boost volume will be randomised to A vs B vs C2 vs D2; all other patients to either A vs B or A vs B vs C1 vs D1. In summary, a suitable boost volume is defined as:

On the pre-biopsy staging multiparametric MRI scan, a dominant intra-prostatic lesion (DIL) has:

- A score 4 or 5 lesion (clinically significant cancer is likely or highly likely to be present) according to the (PI-RADS (v.2) guidelines. Both T2 and DWI are important and this depends on tumour location in the gland.
- DIL with a minimum dimension of 5mm.
- Total DIL volume must be <50% total prostate volume. If there are 2 or 3 DILs, add the individual volumes. Volumes can be estimated with measurement of dimension in 3 directions.

3. PROCEDURES BEFORE PLANNING

3.1. Staging MRI Imaging

Minimum requirement for arms A and B: Standard multiparametric MRI examination with DWI after biopsy and/or starting Androgen Deprivation Therapy (ADT) is acceptable, but less than 2 months ADT duration is <u>desirable</u>.

Minimal requirements for arms C and D: It is recommended to use the trial MRI imaging acquisition protocol (see APPENDIX A) with the minimum dataset for multiparametric MRI acquisition. It is mandatory for arms C2 and D2 to perform the MRI scan pre-biopsy with at least one functional (DWI) image sequence.

<u>PET CT scan:</u> Whole Body MRI, PSMA or choline PET CT scans for staging instead of a bone scan are allowed.

3.2. Androgen Deprivation Therapy (ADT)

Recommended is a LHRH antagonist or LHRH agonist with flare cover. Bicalutamide monotherapy is acceptable if maintenance of sexual potency is a priority. Methods to reduce the risks of breast related side effects are recommended. Total duration of hormone therapy is recommended for 6 months for intermediate risk patients and 2 years for high risk patients and should be specified at registration.
Minimum: planning at month 4-5, duration of ADT up to 6 months before starting radiotherapy.
Optimal: planning at month 2, start RT during month 3

3.3. Image Guidance (IGRT)

- All patients will have daily online image-guided radiotherapy (IGRT)

3.3.1. Use of fiducials:

- Fiducial markers are mandatory for patients in all arms if daily planar online image verification and correction is being used.
- Fiducial markers are **recommended** (but not mandatory) if daily imaging and online correction is with soft tissue imaging, e.g. CBCT, US, CT on rails, tomotherapy or MR-guided RT, and if this is the standard technique and sufficient experience exists in the trial centre (to be agreed with RTTQA team).

Fiducial Placement:

- Fiducial markers (measuring 3-5 mm) should be visible on CT and MRI imaging, if MR-planning, to allow image guidance and MRI/CT fusion. At least three fiducial markers will be placed under transrectal ultrasound guidance, using either transperineal or transrectal approach. Antibiotic cover should be administered if fiducial placement is done transrectally. The physician will place seeds such that they are visible (and not superimposed) on orthogonal imaging (where used) and ideally are separated by 2 cm or more. It is recommended that three seeds for kV imaging and at least two seeds for CBCT or CT imaging are usable for tracking during treatment.
- To allow fiducial stabilisation and resolution of swelling, planning studies will be imaged at least 7 days after fiducial placement.
- If only one fiducial can be seen at planning, either use daily CBCT or insert additional fiducials and re-plan the patient.

4. PLANNING CT AND MRI SCANS FOR EXTERNAL BEAM RADIOTHERAPY

4.1. Patient Preparation and Positioning

4.1.1. Bowel preparation

Bowel preparation is strongly recommended to reduce the rectal diameter for all patients receiving radiotherapy. Aim for a maximum rectal AP diameter of 4 cm, measured at the mid-point of the prostate. It is recommended, but not mandatory, to use mini-enemas which should be used for planning and at least for the initial part of the treatment.

4.1.2. Bladder preparation

It is recommended that patients have a partially filled bladder (150-250 ml) during imaging and treatment delivery: patients should be asked to empty their bladder and then drink enough water (e.g. 325 ml) to ensure a reasonably filled bladder on the planning scan and before each fraction of radiotherapy. If the bladder volume is <150 ml, proceed with planning, check the bladder volume with CBCT during treatment and encourage good hydration.

4.1.3. Immobilisation

All patients will be scanned and treated supine with arms displaced out of the radiotherapy field, using appropriate immobilisation techniques indexed to the treatment couch (e.g. knee and ankle supports). If a planning MRI scan is performed, the same immobilisation is used.

4.2. Planning Scans

4.2.1. Planning CT scan

All patients normally undergo a planning CT scan. A planning MRI only is acceptable, providing this has been agreed with RTTQA physicists. If the anterior-posterior diameter of the rectum is >4 cm at any level adjacent to the prostate, the patient should be rescanned after administration of laxatives. For patients who have a rectum >4 cm despite re-scanning, acquire CBCT at fraction 1 to confirm the rectal size. Patients who are randomised to pelvic node radiotherapy (arms B, D1, D2) should be scanned with IV contrast to aid delineation; in addition, those with limited intra-abdominal fat may be considered for oral contrast administration. The CT scan range must encompass the PTVs and OARs with a margin for dose fall-off. As a guide, go from the top of L4 vertebra to the penile urethra (usually 1 cm below ischial tuberosities will be adequate). CT scans will be taken at ≤3 mm intervals (≤3 mm slice thickness).

4.2.2. Planning MRI scan

In the focal boost IMRT arm (C2, D2), **i**t is strongly recommended that all patients undergo MRI imaging for radiotherapy planning purposes to determine the anatomical borders of the prostate, boost volume and, if possible, the urethra. No endorectal coil is allowed. If the scans are performed on the same day as the CT planning scan, we recommend performing the MRI scan first, but as close together as possible. If the bladder is >200 ml on the initial scan and/or there is a significant delay with the subsequent scan, ask the patient to pass urine to prevent an overfilling of the bladder.

If the scans are not performed on the same day, any sequence is acceptable but the same preparation protocol should be repeated. A urethral catheter is helpful to aid the outlining of the urethra and aid the fusion, but is not mandatory. The MRI will be fused to the treatment planning CT by matching the implanted fiducials, where present. MRI only planning is acceptable.

4.2.3. Planning MRI scan parameters

Perform a scan that covers the prostate and seminal vesicles, normally superiorly above the acetabulum to inferiorly below the penile urethra.

The MRI scan must include a T2-weighted (T2W) transverse series as follows:

- Based on Fast or Turbo Spin Echo FSE/TSE
- Small field of view (FOV), approx. 220 mm
- Matrix 256 (or larger)
- Slice thickness and slice interval 3 mm (or smaller), this should match the planning CT scan
- About 40 slices, providing a coverage of about 120 mm in the superior-inferior direction
- If fiducial markers are not visible on the T2W images, either (on a Philips scanner) reduce the Water-Fat Shift (WFS) to 1.0-1.3 mm or, on other scanners, this is achieved by increasing the Receiver Bandwidth.
- No angulation of slices about the RL or AP axes.

Even if the patient stays nearly motionless, fiducial markers can be difficult to see on the T2W series. Therefore, also perform a Gradient Echo (GE) based scan (e.g. a T2*W) using the same FOV, slice

interval and number of slices as the T2W. The positions of the fiducial markers should be clear on the GE-based scan because of signal-void artefact.

4.2.4. Planning PET CT

If PET CT is used for boost volume definition for focal IMRT, it is important to schedule the scan before ADT as otherwise the intra-prostatic uptake is reduced and less obvious. Use the same CT protocol as for the planning CT to facilitate fusion with the planning CT scan.

4.2.5. Image registration

The planning MRI images are registered to the planning CT scan (if used). The planning CT is the primary dataset. The MRI is registered to it principally using the fiducial markers. If the fiducial markers are difficult to see on the MRI T2W, the registration parameters from an additional MRI T1 or MRI GE sequence can be used. If a PET CT scan is performed, register the CT scan component of the PET with the planning CT scan (primary data set) and use the same registration parameters for the PET component.

5. ORGANS AT RISK AND TARGET VOLUME DEFINITIONS FOR EXTERNAL BEAM PLANNING

5.1. Organs at Risk (OAR)

Organ at risk (OAR) structures and naming convention for PIVOTALboost to be outlined include:

Table 5-1 OARs to be outlined

Volume	Naming convention
Rectum, Rectum plus 2 mm margin	Rectum, Rectum_02
Bladder	Bladder
Urethra	Urethra
Right femoral head	FemoralHead_R
Left femoral head	FemoralHead_L
Penile bulb	PenileBulb
Bowel, Bowel with a isotropic 3 mm margin	Bowel, Bowel_03

5.1.1. OAR definitions

Bladder: The outside of the bladder wall should be outlined. The entire bladder should be included.

Rectum: The circumference of the rectum should be outlined in its entirety. The rectum is outlined to include the rectal contents. Outlining should extend from the bottom of the ischial tuberosities or the anal margin (whichever is inferior) to the recto-sigmoid junction. The recto-sigmoid junction will be defined as the level at which there is an anterior inflection of the bowel – this is usually best appreciated on sagittal reconstructions on the CT planning scan.

Bowel: The individual bowel loops visible on relevant levels of the planning scan will be outlined and will be used for the 'bowel' dose-volume constraint. The outlining will include the small bowel, the large bowel and the sigmoid colon, down to the level of the recto-sigmoid junction. The superior extent of outlining should be 2 cm beyond the superior extent of CTVpsv or CTVn as appropriate.

During the outlining for CTVn, ‘bowel’ is expanded by an isotropic 3 mm margin to create an outlining assistance volume ‘Bowel_03’; this structure is used as an avoidance structure and is excluded from CTVn, whether generated from vessels or manually.

Right and Left femoral heads: The femoral heads are outlined to the bottom of the curvature of their heads (femoral necks are not included).

Urethra: Outline the structure only if it is visible on the planning scan. Outline the urethra or catheter from the inferior to superior end of the prostate PTV; use the MRI to locate the structure.

Penile bulb: Use the MRI scan as guidance to outline the structure and then adapt to location on CT scan. The volume is normally 9-10 mm in superior-inferior direction.

5.2. Target Volume Definition

Volumes will be defined according to ICRU reports 50, 62 and 83 [1.2.3]. Outlining should be carried out with the aid of the diagnostic and planning MRI in accordance with the outlining protocol (see below and supplementary files: “PIVOTALboost Boost Contouring Atlas” and “PIVOTALboost pelvic node Contouring Atlas”). For all arms, the prostate target and lymph node volumes are outlined in the same way.

Use the exact nomenclature below.

5.3. GTV and CTV Definition

Table 5-2 Definition of GTV and CTVs

Volume	Structure	Definition
GTVpb	Boost volume	Tumour volume on the staging mpMRI
CTVpb		GTVpb + 3 mm, except not extending outside CTVpsv
CTVp	Prostate	Prostate, plus proximal centre of seminal vesicles, 1 cm above the origin and any extraprostatic extension (periprostatic fat, seminal vesicle or base of bladder). If the seminal vesicles are short, include the appropriate length
CTVpsv	Prostate and seminal vesicles	CTVp and any remaining seminal vesicle
VESSEL	Pelvic vessel	Left- and right-sided external iliac, internal iliac and obturator vessels.
CTVn	Pelvic nodes	Pelvic nodal volume

5.4. Prostate Volumes

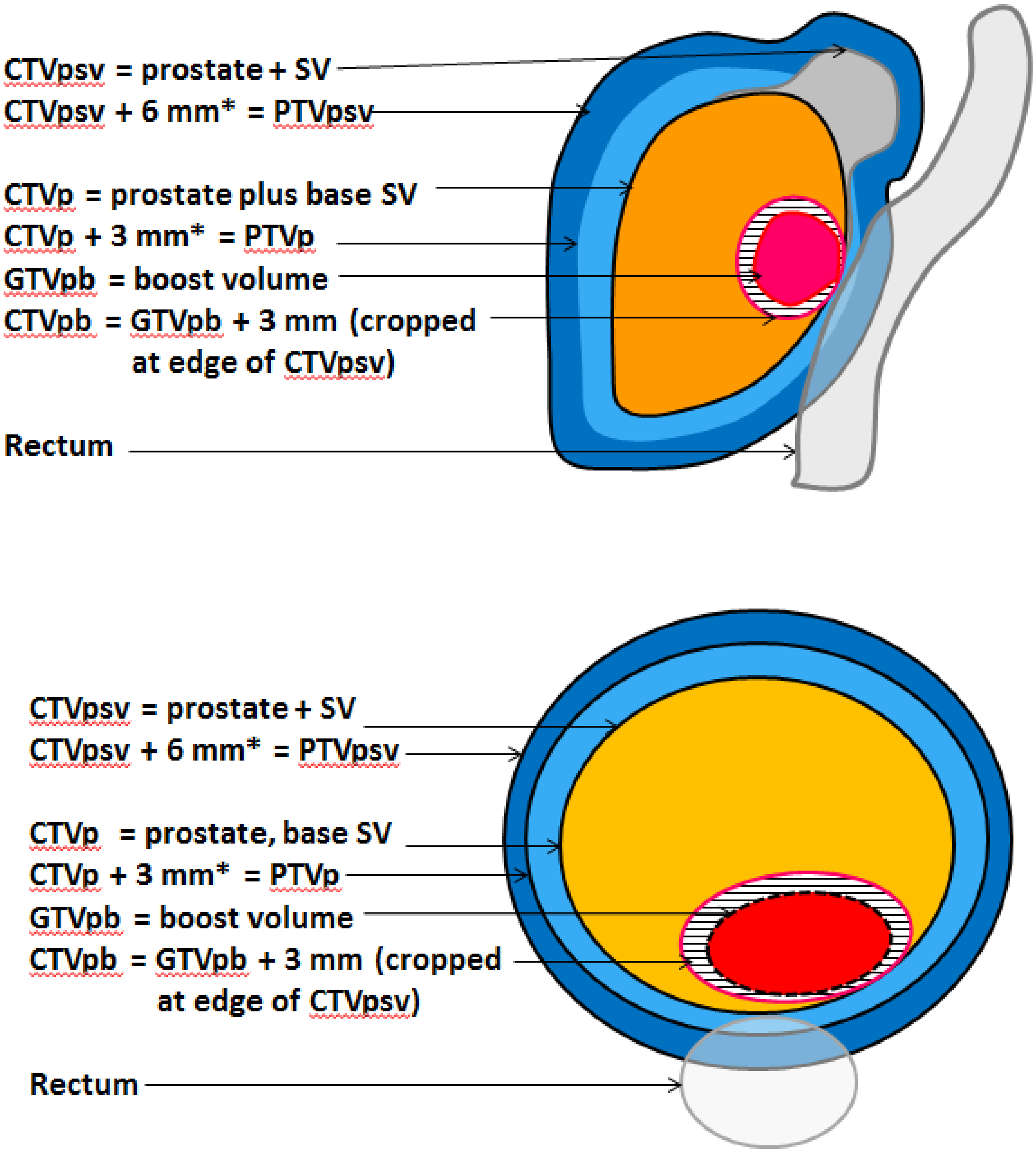

** Note that margins are 8mm and 4mm if fiducials are not present*

Figure 5.1 Sagittal and Transverse diagrammatic representation of GTVs and CTVs

5.5. Clinical Target Volumes and Planning Target Volumes (for External Beam RT)

Table 5-3: Arms A and B: prostate and prostate with pelvic node (PPN) IMRT

Arm	Site	Structure name	Definition
**A**	Prostate + SV	CTVpsv<br>PTVpsv_4700	Prostate and seminal vesicles<br>With fiducials: CTVpsv plus 6 mm<br>Without fiducials: CTVpsv plus 8 mm
	Prostate	CTVp<br>PTVp_6000	Prostate, base of seminal vesicles<br>With fiducials: CTVp plus 3 mm<br>Without fiducials: CTVp plus 4 mm
**B**	Prostate+ SV	CTVpsv<br>PTVpsv_4700	Prostate and seminal vesicles<br>With fiducials: CTVpsv plus 6 mm<br>Without fiducials: CTVpsv plus 8 mm
	Prostate	CTVp<br>PTVp_6000	Prostate, base of seminal vesicles<br>With fiducials: CTVp plus 3 mm<br>Without fiducials: CTVp plus 4 mm
	PPN	CTVn<br>PTVn_4700	Pelvic lymph nodes<br>CTVn plus 5 mm

Table 5-4: Arms C1, D1, C2 and D2 external beam radiotherapy with HDR

Arm	Site	Structure name	Definition
**C1 & C2**	Prostate + SV	CTVpsv<br>PTVpsv_3750	Prostate and seminal vesicles<br>With fiducials: CTVpsv plus 6 mm<br>Without fiducials: CTVpsv plus 8 mm
**D1 & D2**	Prostate + SV	CTVpsv<br>PTVpsv_4200	Prostate and seminal vesicles<br>With fiducials: CTVpsv plus 6 mm<br>Without fiducials: CTVpsv plus 8 mm
	PPN	CTVn<br>PTVn_4700	Pelvic nodes<br>CTVn plus 5 mm

Table 5-5: Arms C2 and D2 focal boost IMRT

Arm	Site	Structure name	Definition
**C2**	Prostate + SV	CTVpsv<br>PTVpsv_4700	Prostate and seminal vesicles<br>With fiducials: CTVpsv plus 6 mm<br>Without fiducials: CTVpsv plus 8 mm
	Prostate	CTVp<br>PTVp_6000	Prostate, base of seminal vesicles<br>With fiducials: CTVp plus 3 mm<br>Without fiducials: CTVp plus 4 mm
	Boost	GTVpb1, GTVpb2<br>CTVpb	Outline boost volume(s)<br>GTVpb1 +3 mm, GTVpb2 +3 mm margin + …<br>All volumes form the CTVpb, with no extension outside CTVpsv

**D2**	Prostate + SV	CTVpsv<br>PTVpsv_4700	Prostate and seminal vesicles<br>With fiducials: CTVpsv plus 6 mm<br>Without fiducials: CTVpsv plus 8 mm
	Prostate	CTVp<br>PTVp_6000	Prostate, base of seminal vesicles<br>With fiducials: CTVp plus 3 mm<br>Without fiducials: CTVp plus 4 mm
	Boost	GTVpb1, GTVpb2<br>CTVpb	Outline boost volume(s)<br>GTVpb1 +3 mm, GTVpb2 +3 mm margin + …<br>All volumes form the CTVpb, with no extension outside CTVpsv
	PPN	CTVn<br>PTVn_4700	Pelvic nodes<br>CTVn plus 5 mm

6. EXTERNAL BEAM RADIOTHERAPY PLANNING GUIDELINES

6.1. Radiotherapy Technique

This trial simultaneously treats multiple dose-level PTV structures in a single phase. Therefore, an IMRT or VMAT planning technique must be used to obtain the prescription doses for adjacent PTVs. Pelvic lymph node treatments are randomly allocated to patients in the trial.

6.2. Prescribed Dose and Fractionation

Arms A and B treat the patient in 20 fractions. Both arms treat the prostate to 60 Gy and the seminal vesicles to 47 Gy, with Arm B also simultaneously treating the pelvic lymph nodes to 47 Gy in 20 fractions.

Non-HDR Arms C2 and D2 treat the patient in 20 fractions. Building upon Arms A and B, they add a focal boost to the prostate GTV, prescribing 67 Gy to this boost volume. Arm C2 follows the prescriptions in Arm A, treating the prostate and seminal vesicles, whilst Arm D2 follows the prescriptions in Arm B, treating the prostate, seminal vesicles and pelvic lymph nodes.

The HDR arms have the external beam treatment boosted by HDR therapy. For those patients where a prostate GTV volume is to be boosted, this will be done during the brachytherapy treatment.

HDR Arms C1 and C2 treat the prostate and seminal vesicles as a single structure receiving 37.5 Gy in 15 fractions for the external beam portion of treatment.

HDR Arms D1 and D2 treat the patient in 20 fractions. For the external beam portion of treatment the prostate and seminal vesicles are treated as a single structure receiving 42 Gy, whilst the pelvic lymph nodes receive 47 Gy.

The PTVs must receive a minimum of 95% of the prescription dose to 98% of the volume (see Table 6-1 to Table 6-3). If the PTVs overlap with any OARs or PRVs try to minimise the dose in the overlap region whilst maintaining a minimum PTV coverage of 95% dose (see Figure 6.1 below for guidance).

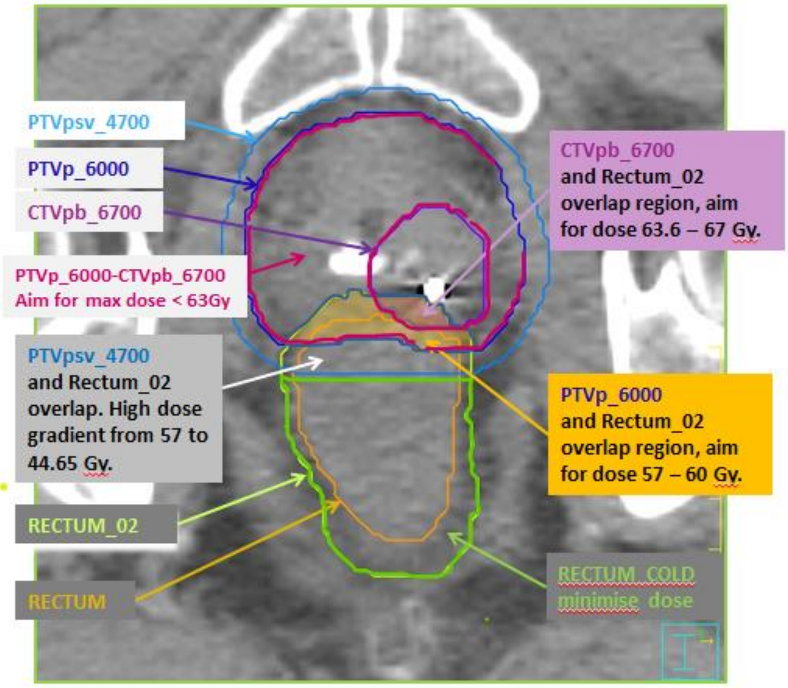

Figure 6.1: Planning goals (i.e. aim of optimisation) for the PIVOTALboost trial

6.3. Definition of PTVs for Dose Reporting with their Dose Constraints

Table 6-1: Arms A and B prostate and prostate with pelvic node IMRT

Planning Target Volumes	Dose to PTV
Prostate + SVs PTV	Report dose to PTVpsv_4700–PTVp_6000<br>$D_{50\%}$ ≥ 47.0 Gy (median**); $D_{98\%}$ ≥ 44.65 Gy (95%)
Prostate-only PTV	Report dose to PTVp_6000<br>$D_{50\%}$ = 60 Gy (median**); $D_{98\%}$ ≥ 57.0 Gy (95%), $D_{2\%}$ ≤ 64.2 Gy (107%)
Pelvic Nodes PTV	Report dose to PTVn_4700–PTVpsv_4700<br>$D_{50\%}$ =47 Gy (median**); $D_{98\%}$ ≥ 44.65 Gy (95%), $D_{2\%}$ ≤50.3 Gy (107%)

Table 6-2: Arms C1, D1, C2 and D2 external beam radiotherapy with HDR

**Prostate and seminal vesicles only (C1, C2)**	
Prostate + SVs PTV	Report dose to PTVpsv_3750<br>$D_{50\%}$ = 37.5 Gy (median**); $D_{98\%}$ ≥ 35.6 Gy (95 %); $D_{2\%}$ ≤ 40.1 Gy (107 %)
**Prostate, seminal vesicles and pelvic nodes (D1, D2)**	
Prostate + SVs PTV	Report dose to PTVpsv_4200–PTVn_4700<br>$D_{50\%}$ ≥ 42.0 Gy (median**); $D_{98\%}$ ≥ 39.9 Gy (95%); $D_{2\%}$ ≤ 44.9 Gy (107%)
Pelvic Nodes PTV	Report dose to PTVn_4700<br>$D_{50\%}$ = 47 Gy (median**); $D_{98\%}$ ≥ 44.65 Gy (95 %); $D_{2\%}$ ≤50.3 Gy (107%)

Table 6-3: Arms C2 and D2 focal boost with IMRT

Planning Target Volumes	Dose to PTV
Prostate + SVs PTV	Report dose to PTVpsv_4700–PTVp_6000<br>$D_{50\%} \geq 47.0$ Gy (median**); $D_{98\%} \geq 44.65$ Gy (95%)
Prostate-only PTV	Report dose to PTVp_6000<br>60.0 Gy $\leq D_{50\%} \leq$ 63.0 Gy (median**); $D_{98\%} \geq 57.0$ Gy (95%)
Focal Boost CTV	Report dose to CTVpb<br>†$D_{50\%}$ = 67.0 Gy† (median**), $D_{2\%} \leq 70.0$ Gy
Pelvic Nodes PTV	Report dose to PTVn_4700–PTVpsv_4700<br>$D_{50\%}$ = 47 Gy (median**); $D_{98\%} \geq 44.65$ Gy (95%); $D_{2\%} \leq 50.3$ Gy (107%)

*** For median doses, values within 1% of the stated figure are acceptable.*
*† The median dose may be reduced (60.0 Gy ≤ $D_{50\%}$ ≤ 67.0 Gy) if CTVpb > 0.5*CTVp*

6.4. Normal Tissue Dose Constraints for Organs at Risk for External Beam Plans

(Note that Brachytherapy dose constraints are in Section 7.4)

The nomenclature used in Section 5.1 Organs at Risk (OAR) should be used for planning in all benchmark and clinical cases. Failure to do so may result in the plan being returned.

Note that OARs have both **optimal** dose constraints that should be the aim in planning and **mandatory** constraints that must be achieved for the plan to be acceptable. When reporting, ensure that the entire OAR volume, including any overlap with the PTVs, has been included.

Aim to reduce the dose in the PRV structures but report the doses to the OAR structures.

Table 6-4: Arms A, B, C2, D2 external beam radiotherapy without HDR

Organ	Dose for 20# [Gy]	Maximum Volume	
		Optimal	Mandatory
Rectum*	24	80 %	-
	32	65 %	-
	40	50 %	60 %
	48	35 %	50 %
	52	-	30 %
	56	-	15 %
	60	3 %	5 %**
	64	0 %	1 %**
	67	0 %	0 %
		$D_{mean} \leq 35$ Gy	
Bladder	40	50 %	-
	48	25 %	50 %
	60	5 %	35 %
Bowel#	36	78 cm^3	158 cm^3

	40<br>44<br>48<br>52	17 cm^3<br>14 cm^3<br>0.5 cm^3<br>0 cm^3	70 cm^3<br>28 cm^3<br>6 cm^3<br>0 cm^3
Penile bulb	40	50 %	-
	48	10 %	-
Left Femoral Head	40	5 %	50 %
Right Femoral Head	40	5 %	50 %

**The rectal mean dose of 35 Gy should not compromise the coverage of PTVp_6000 or PTVpsv_4700. If $D_{3\%} \leq 60$ Gy and $D_{mean} \leq 35$ Gy the rectal NTCP for rectal bleeding and faecal incontinence do not exceed 6 %.*
# If the mandatory dose constraints are not achieved and the plan is optimal, the coverage of PTVn_4700 should be reduced to achieve the dose constraints.
*** Coverage of CTVpb may be compromised if adjacent to Rectum.*

Table 6-5: Arms C1, D1, C2 and D2 external beam radiotherapy with HDR

Organ	Dose [Gy]	Maximum Volume	
		Optimal	Mandatory
**Prostate only (15# treatment, C1, C2)**			
Rectum	25<br>30<br>33<br>36<br>37.5	50 %<br>35 %<br>-<br>-<br>3 %	60 %<br>50 %<br>30 %<br>15 %<br>5 %
Bowel	36	78 cm^3	158 cm^3
Bladder	25	50 %	-
**Prostate and pelvic nodes (20# treatment, D1, D2)**			
Rectum	31<br>37<br>41<br>44<br>47	50 %<br>35 %<br>-<br>-<br>3 %	60 %<br>50 %<br>30 %<br>15 %<br>5 %
Bowel	36<br>40<br>44<br>48	78 cm^3<br>17 cm^3<br>14 cm^3<br>0.5 cm^3	158 cm^3<br>70 cm^3<br>28 cm^3<br>6 cm^3
Bladder	40	50 %	-

For dose calculation, a Type B algorithm is preferred but a Type A would be acceptable. The dose grid must encompass the PTV and all the OARs and have a maximum resolution of 2.5 mm.

6.5. Extra Considerations concerning Treatment Delivery

For centres carrying out VMAT with only one machine capable of delivery, a backup fixed field IMRT plan must be produced and submitted as part of the planning pre-trial QA.

For centres intending to deliver with different modalities, e.g. Tomotherapy and Linac, benchmark plans for all modalities must be produced and submitted as part of the planning pre-trial QA.

7. WHOLE GLAND HDR and FOCAL HDR: TARGET VOLUMES FOR ARMS C1, D1, C2 AND D2

Table 7-1: Brachytherapy Structures

Structure	Description
CTVp	Prostate and any extra-capsular extension or SV involvement plus 3 mm 3D expansion constrained posteriorly to the anterior rectal wall and superiorly to the bladder base
CTVpb	Define EITHER<br>by contouring GTVpb on the staging MRI scan. Add 3 mm 3D expansion for the CTVpb, except not extending beyond CTVp. The volume can be transferred by cognitive fusion or fusion with the planning CT/MRI or planning ultrasound.<br>OR<br>by identifying involved segments from staging MRI scan. 12 prostate sectors are defined by first dividing into three base, mid-gland and apex segments, and then dividing each of these into four sectors: right anterior, left anterior, right posterior and left posterior. The sectors intersected by the F- GTV/F-PTVs are manually determined. No additional margin is added, but the segment volume does not extend into the urethra or beyond CTVp

Table 7-2: Clinical Target Volumes

Arm	Modality	Structure name	Definition
**C1**	HDR	CTVp_1500	Prostate, base SV + 3 mm expansion
**D1**	HDR	CTVp_1500	Prostate, base SV + 3 mm expansion
**C2**	HDR	CTVp_1500	Prostate, base SV + 3 mm expansion
		CTVpb_1900	Boost volume does not extend into the urethra
**D2**	HDR	CTVp_1500	Prostate, base SV + 3 mm expansion
		CTVpb_1900	Boost volume does not extend into the urethra

Table 7-3: PTV Dose Prescription

PTV	Dose to PTV
**Brachytherapy**	
CTVp_1500	15 Gy minimum peripheral dose (100%)<br>$D_{90\%}$≥100%; V_{15Gy}≥95 % for CTVp_1500
CTVpb_1900	Aim for $D_{90\%}$≥19 Gy; V_{19Gy}≥90% for CTVpb_1900 respecting OAR constraints (urethra and rectum)

Table 7-4: Normal Tissue Dose Constraints for HDR

Organ	Dose constraint
Rectum	D_{2cc} ≤11.8 Gy<br>$V_{100\%}$ (V_{15Gy}) = 0%
Urethra	$D_{10\%}$ ≤17.5 Gy<br>$D_{30\%}$ <16.5 Gy<br>$V_{150\%}$ ($V_{22.5Gy}$) = 0%

High dose rate brachytherapy will be performed according to the centre's usual technique. Transrectal ultrasound based outlining and planning, (one stage procedure) and CT/MRI planning is acceptable. The CTV will be defined as detailed in the GEC-ESTRO guidelines [Hoskin et al] to conform to the CTVp_1500 which includes the prostate capsule and is expanded to include any known areas of extracapsular extension or seminal vesicle involvement identified on pre-treatment staging scans with a 3 mm expansion constrained to the outer rectal wall.

The CTVp_1500 for focal HDR boost is the same as for the whole gland HDR. The boost volume includes the volume identified on pre-treatment staging scans. Boost volumes can be defined by contouring or by sector optimisation [Mason et al]. In sector optimisation the prostate is divided into anatomical sectors and those sectors involved with tumour on pre-treatment imaging are then boosted without additional margins. For focal boost HDR, normal tissue constraints (rectum and urethra) might limit the dose to CTVpb_1900. Boost dose may be reduced to ensure rectal and urethral dose constraints are not exceeded. The GTVpb volume should be <50% of the CTVp_1500. To achieve a boost dose >16 Gy and adhere to the normal tissue constraints, it is recommended to boost only a maximum of 3 different CTVpb, and / or no more than 6 sectors.

7.1. Brachytherapy Reporting Parameters

The following parameters will be reported:

- Implant technique and number of catheters
- Total reference air kerma (TRAK). Total source exposure
- CTVp_1500: D90%, V100%, V150%, V200%
- CTVpb_1900: D90%, V100%, V150%, V200%
- OAR Rectum: D2cc, V100%
- OAR Urethra: D10%, D30%, V150%

8. RADIOTHERAPY DELIVERY

It is important to ensure that patients follow the bladder/bowel preparation instructions they have used at the planning CT scan appointment.

8.1. IGRT (please also refer to APPENDIX B. GENERAL GUIDELINES FOR IGRT)

All patients will have daily image-guided radiotherapy. It is recommended that all patients be set up prior to treatment and if a significant shift is required (> 10 mm) the patient should be re-imaged after that shift. CBCT, Tomotherapy, and ultrasound are acceptable if this is a standard protocol in the centre.

8.2. Fiducial Markers and kV Imaging

Patients should be imaged daily using planar kV images and a 2 mm tolerance. At least two fiducials should be identified for each treatment. If only one fiducial can be tracked, CBCT should be used. Where the ability exists, rotational corrections should be made. If there are shifts > 10 mm, CBCT imaging pre-treatment is recommended (mandatory for focal IMRT patients) to rule out any significant changes in rectal position or prostate deformation. For focal IMRT plans, the dose distribution should be assessed before the next fraction. The markers should be checked for positioning errors.

8.3. Cross Sectional Imaging (CBCT, CT on Rails, Tomotherapy)

Patients should be imaged daily using CBCT. The fiducial markers (if available) will be used as the reference for prostate position and all shifts should be applied.

If at fraction 1, any one shift value is greater than 10 mm do not treat until the images can be reviewed. Check the bone match, rectal and bladder filling, if necessary re-position or re-plan. If, during treatment, shifts of > 10 mm are observed in any direction, arrange a review by the clinician.

8.4. Cross Sectional Imaging (Ultrasound)

The use of daily ultrasound image guidance needs to be discussed with the RTQA team before treating any patient in the trial.

9. TREATMENT SCHEDULING

Treatment can start on any day of the week except Monday. The treatment course must not be delivered in less than 28 days. Unscheduled treatment interruptions up to 5 days are acceptable. If further delays have occurred (e.g. machine breakdown), record this and the reasons on the treatment CRF. Do not treat more than one fraction per day and 5 fractions per week.

10. DOCUMENTATION ON COMPLETION OF RADIOTHERAPY

On completion of radiotherapy planning, all plans (including MRI (if available) and CT images, structures, plan and dose matrix) and the Plan Assessment Form (Excel version), should be exported, anonymised and sent to the RTTQA team electronically following the exporting data guidelines in the next section. IGRT displacement data does not need to be collected; please discuss any concerns with the QA team.

11. RADIOTHERAPY QUALITY ASSURANCE

11.1. Radiotherapy Quality Assurance Overview

Radiotherapy QA includes pre-trial and on-trial components.

Pre-trial QA includes (documentation to be downloaded from http://www.rttrialsqa.org.uk):

- Initial survey
- Benchmark outlining cases
- Benchmark planning case
- Outlining workshop / webinar (or reading of presentations from workshop)
- Facility questionnaire

On-trial QA includes:

- Prospective and/or retrospective case reviews
- Review of HDR implant parameters
- Collection of staging MRI imaging
- Dosimetry site visit (subject to prior RTTQA dosimetry accreditation)

All plans (including MRI, CT, structures, plan and dose cube) and the Plan Assessment Form should be exported, anonymised and sent to the QA team electronically.

11.2. Pre-Trial Questionnaires

11.2.1. Initial Survey

To be completed before starting pre-trial QA. The survey collects preliminary information which will be used to inform the QA process. If you have not been sent a survey, please email pivotalboost.trial@nhs.net.

11.2.2. Facility Questionnaire

The Facility Questionnaire collects information about the RT equipment, techniques and procedures used by a centre for the trial. This can be partly substituted by the centre's own work instructions where applicable. The questionnaire will be provided by the PIVOTALboost RTQA team as it may be pre-filled with information provided from previous RTTQA pelvic RT trials.

11.3. Benchmark Cases and downloading date from RTTRIALS website

- Download the planning data (CT or US images and Structure set) from the RTTQA web site: http://www.rttrialsqa.org.uk
- Log in.
- Select 'Downloads' from the menu bar.
- Open 'Top Level Folders' (see below) and select the PIVOTALboost folder.

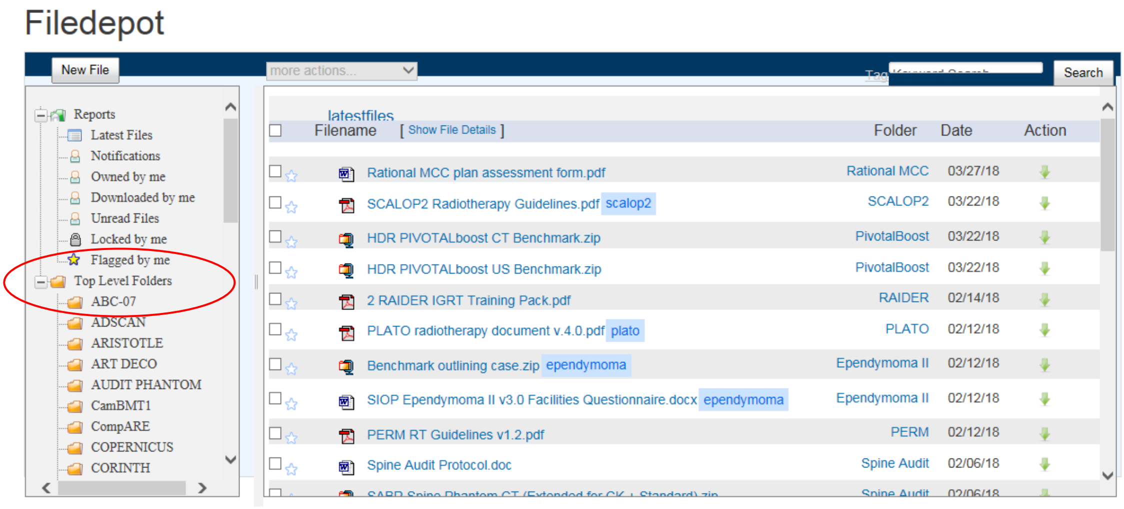

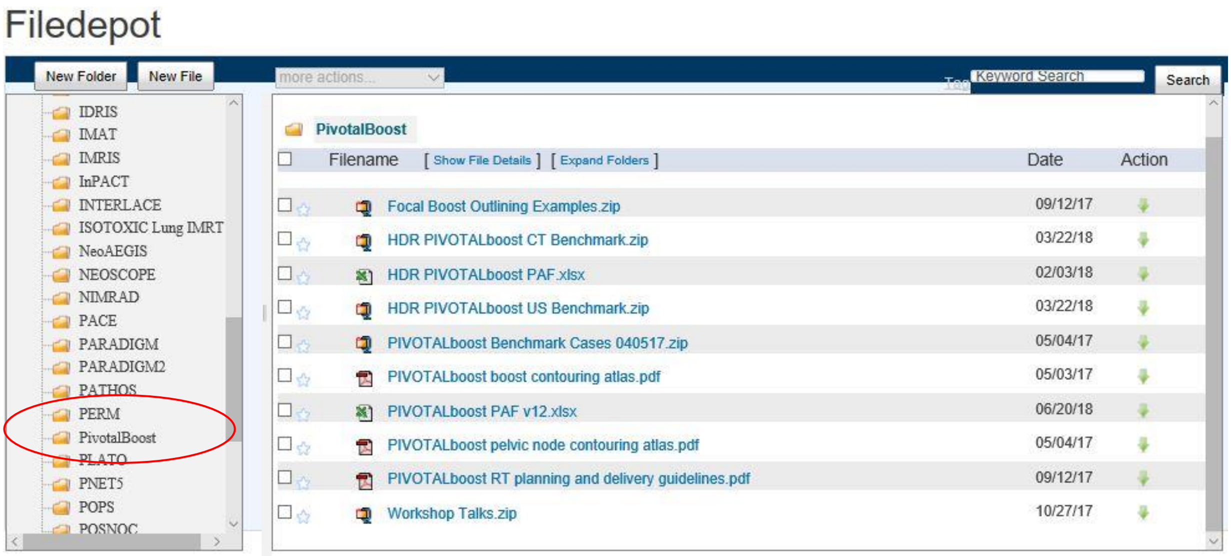

11.4. Outlining Benchmark Cases

All centres wishing to participate in the PIVOTALboost trial will need to complete two contouring exercises. For each case a planning CT, a planning MR, and a PDF file containing the clinical history and snapshots through the prostate on diagnostic MRI scans (from Inf to Sup) are provided. The data should be downloaded from the RTTQA website (see above).

It is not mandatory to use the planning MR, but if you wish to do so you will need to register it to the planning CT. You can use the urethra and the fiducials (small black holes on MR) to help you.

Refer to outlining instructions in Section 5, supplemented by the "PIVOTALboost Boost Contouring Atlas" and the "PIVOTALboost pelvic node Contouring Atlas". Please use the trial structure naming convention.

Once outlines have been created, reviewed and accepted by the local PI, please export and return the DICOM CT and Structure data to the RTQA team using RTTQA data transfer, see section 11.11

11.4.1. Outlining Benchmark Case 1 – Boost volume outlining

Contouring Instructions: Please import planning CT and MRI data sets for name/ID=pivotalboostpat1 into your TPS. The following structures have been pre-outlined:

- CTVp
- Rectum
- Bladder
- FemoralHead_L and _R
- PenileBulb
- Urethra

Outline the boost volume(s), GTVpb, only.

11.4.2. Outlining Benchmark Case 2 – Boost volume, prostate and pelvic nodes outlining

Contouring Instructions: Please import planning CT and MRI data sets for name/ID=pivotalboostpat2 into your TPS. The following structures have been pre-outlined:

- Rectum
- Bladder

- Bowel
- Bowel_03
- PenileBulb
- Urethra

Outline the prostate and seminal vesicles (CTVp and CTVpsv), the boost volume(s), GTVpb, and the pelvic lymph nodes (VESSEL and CTVn).

11.5. Focal Boost Outlining Examples

There are outlining examples on the website which may be used for training.

11.6. Planning Benchmark Case

All trial centres must complete and submit the PIVOTALboost pre-trial planning benchmark case. The CT images and pre-outlined structure set are available for download from the RTTQA website.

Radiotherapy contouring/planning:

Please import the CT images and structure set for name/ID= PIVOTALboostpl into your own TPS system. The CT has been delineated by the CI with the following structures, which should not be edited. No additional structures, e.g. for PTV/OAR overlaps, have been created. The individual centre should create these as needed.

Target volumes: CTVp, CTVpsv, GTVpb, CTVn, PTVp_6000, PTVpsv_4700, CTVpb, PTVn_4700

OARs: Rectum, Bladder, Bowel, BowelBag, FemoralHead_L and _R, PenileBulb, Urethra

PRVs: Rectum_02, Bowel_03

This patient should be planned as a focal boost IMRT case with pelvic nodes (i.e. trial arm D2). N.B. for centres recruiting to arms A and B only (i.e. those not doing an external beam focal boost) please plan as a prostate with pelvic nodes IMRT case (i.e. trial arm B).

Please complete the PIVOTALboost plan assessment form and provide a copy of the treatment planning report from your TPS.

Data Export: Once the benchmark plan has been created and reviewed and accepted by the local PI, the export of the CT images, dose matrix, RT plan and structure set in DICOM format should be returned to the RTTQA team using RTQA data transfer, see section 11.11. Avoid re-anonymising as this causes problems and may delay your review.

Treatment plan Dosimetry Check: QA dosimetry check measurements are only needed for centres where it is the method of checking the dosimetry of all IMRT plans (PDIP, phantom etc.). Please do a measurement and return the result. For other centres, please do a dosimetry check by your usual method and return the result (e.g. Mobius users).

Note that RTQA approval can be obtained when the external beam benchmark cases have been approved. However, you cannot recruit your first patient (be “activated”) until you have returned your Facility Questionnaire and had it approved. In the case of an HDR centre you cannot recruit to HDR until you have had the HDR benchmark case approved.

11.7. HDR Planning Benchmark

Download planning data

- Download the following from www.rttrialsqa.org.uk: HDR Pivotalboost US benchmark.zip, HDR Pivotal boost CT benchmark.zip (US or CT planning data and structure set) and the HDR plan assessment form ('HDR Pivotal Boost PAF').

Import the relevant images and structure set from HDR PIVOTALboost US Benchmark.zip into your TPS system. The image set has been delineated with the following structures:

Target volumes: Prostate, CTVp_1500, CTVpb_1900, GTVpb

OARs: Rectum, Urethra

The CTV has been margined from the prostate using a 3 mm asymmetric margin with a 0 mm posterior margin. If this is consistent with your centre's protocol, continue planning with the structures provided. If you perform a different posterior margining process, please edit the CTVp_1500 accordingly. N.B. Please comment on the margin process used.

This patient should be planned as a focal boost case (i.e. trial arm D2).

Please complete the PIVOTALboost HDR plan assessment form.

11.7.1. Oncentra Prostate

For guidelines on ultrasound-based planning using Oncentra Prostate please see the instructions below:

1. Importing the study

- Open the Smoothbase application.
- Select Import DICOM files.
- In the Open DICOM files dialogue, navigate to the folder containing the files and select all files, hit OK
- Patient with ID ZZPivotal_Boost should be imported and created.

2. Producing a treatment plan

- Open Oncentra Prostate and load the study associated with patient ZZPivotal_Boost
  In the File Operations tab, double-click RTSTRUCT1 to load the ultrasound images and structure set.
- Apply the dose prescription.
- Insert Catheters. Plan the patient as normal for your centre's technique. Additional needles can be added to target the focal boost area. Ignore the needle positions visible in the image set as these may differ from your centre's implant technique.
  To achieve a clinically realistic plan, virtual catheter positions can be adjusted to the positions you would expect them to be for a plan, as follows:
  - Insert virtual catheters as required.
  - Switch to Live Plan mode, the Live Imaging tab will appear.
  - Click on the Catheter Reconstruction tab. Select Manual Reconstruction.
  - Click on each catheter in turn to change it from virtual to live.
  - Enter an appropriate free length for each catheter and lock each free length (the quickest way to do this is to click on the head of the Free Len.(cm) column in the catheter table then enter the free length against any one catheter, hit enter and all catheter free lengths will be updated and locked).
  - Adjust position of each catheter as required

- Activate Sources according to your standard protocol
- Plan optimization: there are two options:

(i) To produce a plan with a boost optimized to CTVpb_1900:

- Click on the Dose Optimization tab
- Click on Dose sampling settings
- Tick CTVpb_1900. Check that the urethra has the lowest priority value and CTVpb_1500 has the highest priority value, as shown in the following image.

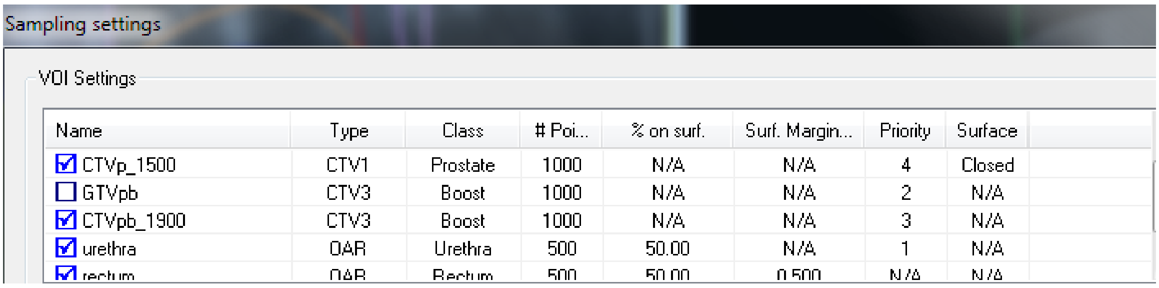

Sampling settings

VOI Settings

Name	Type	Class	# Poi...	% on surf.	Surf. Margin...	Priority	Surface
☑ CTVp_1500	CTV1	Prostate	1000	N/A	N/A	4	Closed
☐ GTVpb	CTV3	Boost	1000	N/A	N/A	2	N/A
☑ CTVpb_1900	CTV3	Boost	1000	N/A	N/A	3	N/A
☑ urethra	OAR	Urethra	500	50.00	N/A	1	N/A
☑ rectum	OAR	Rectum	500	50.00	0.500	N/A	N/A

- Click on Optimization Settings. Alter the values to match your usual settings, with dose limits and Imp. factors set for CTVpb_1900 to achieve the focal boost, as shown in the following image.

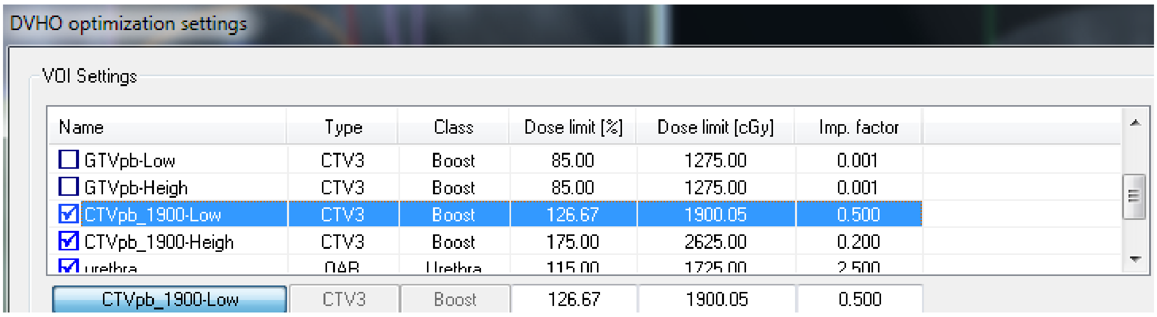

DVHO optimization settings

VOI Settings

Name	Type	Class	Dose limit [%]	Dose limit [cGy]	Imp. factor
☐ GTVpb-Low	CTV3	Boost	85.00	1275.00	0.001
☐ GTVpb-Heigh	CTV3	Boost	85.00	1275.00	0.001
☑ CTVpb_1900-Low	CTV3	Boost	126.67	1900.05	0.500
☑ CTVpb_1900-Heigh	CTV3	Boost	175.00	2625.00	0.200
☑ urethra	OAR	Urethra	115.00	1725.00	2.500
CTVpb_1900-Low	CTV3	Boost	126.67	1900.05	0.500

- Optimize. Evaluate the resulting plan and adjust CTVpb_1900 optimization settings (or use manual dwell time editing/graphical optimization) as appropriate to achieve an acceptable plan.

(ii) To produce a plan with boost optimized by sectors:

- Switch to the VOI handling tab.
- Click on prostate model.
- Tick 'Use models on CTV1' and select the correct geometric model to define sectors as stated in the trial protocol, as shown in the image below.
- For each sector that is being boosted, the Optimisation and Evaluation entries should be set to Yes. For other sectors, these should be set to No. Values can be changed by double clicking. Double clicking the sector name navigates to the centre of the sector.

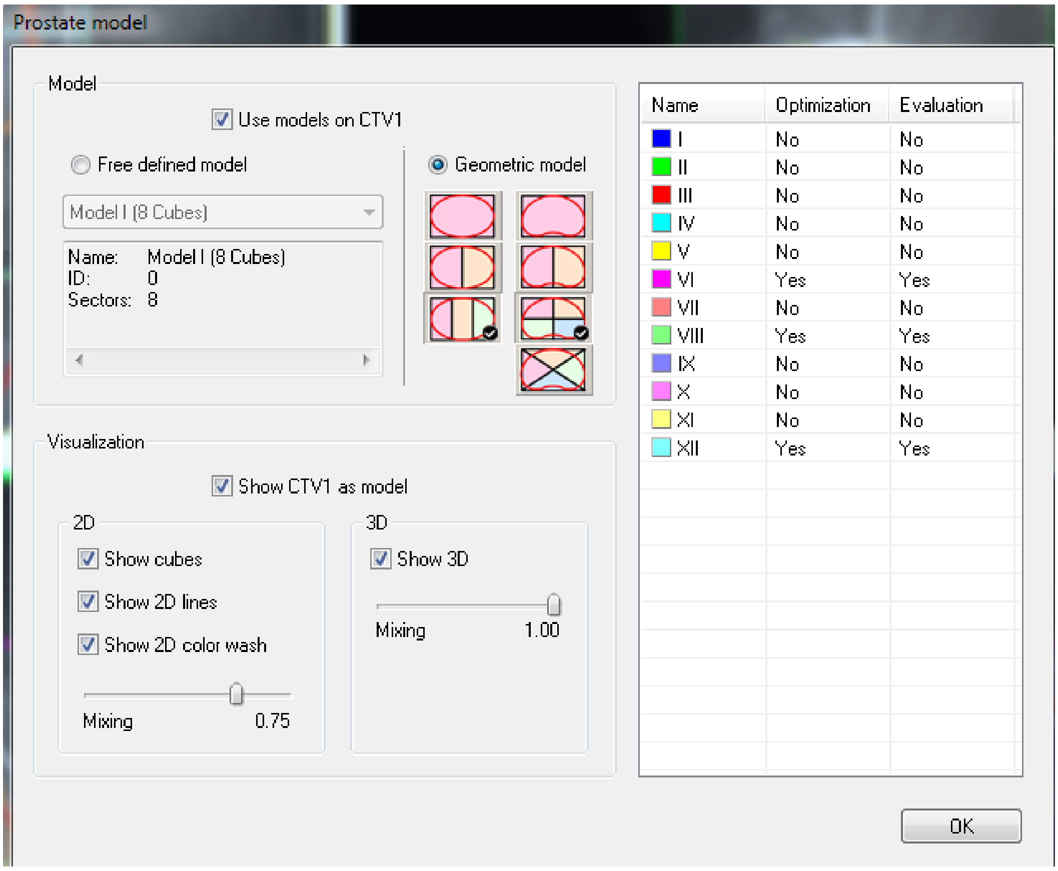

- Hit OK to close the dialogue and switch to the Dose Optimization tab.
- Click on Optimization Settings. Alter the values to match your usual settings, with dose limits and Imp. factors set for the sectors to achieve the focal boost, as shown in the following image.

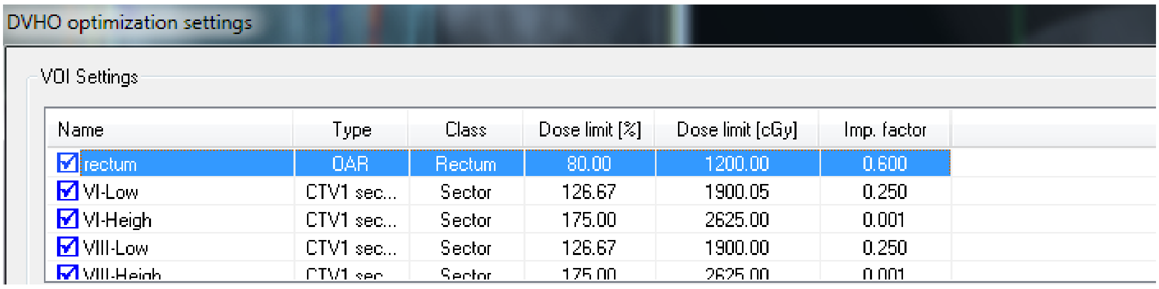

DVHO optimization settings

VOI Settings

Name	Type	Class	Dose limit [%]	Dose limit [cGy]	Imp. factor
rectum	OAR	Rectum	80.00	1200.00	0.600
VI-Low	CTV1 sec...	Sector	126.67	1900.05	0.250
VI-Heigh	CTV1 sec...	Sector	175.00	2625.00	0.001
VIII-Low	CTV1 sec...	Sector	126.67	1900.00	0.250
VIII-Heigh	CTV1 sec	Sector	175.00	2625.00	0.001

Optimize. Evaluate the resulting plan and adjust sector optimisation settings (or use manual dwell time editing/graphical optimisation) as appropriate to achieve an acceptable plan.

3. Planning guidelines

The boost dose achieved will be limited by the OAR dose constraints and requirement to meet the whole gland coverage objectives. When planning this case, the first priority is to meet the OAR dose constraints, the second priority is to achieve acceptable whole gland coverage and the third priority is the boost dose. The urethra D30 < 16.5 Gy can be considered a soft constraint and higher D30 is allowed as long as the D10 < 17.5 Gy is achieved.

The PIVOTAL boost dose aims of CTVpb_1900 D90 ≥ 19 Gy; V19 Gy ≥ 90 % are not likely to be achieved for large volume boosts, or boosts involving the anterior part of the gland due to urethral dose constraints.

In this specific case ZZPivotal_Boost

- when planning with boost optimized to CTVpb_1900 it is possible to achieve V19 Gy ≥ 90 % although this may require the urethra soft constraint D30 < 16.5 Gy to be exceeded.
- when optimizing using sectors, it is possible to achieve V19 Gy ≥ 90% in the posterior sector at the base of the prostate, however a lower V19 Gy will be achieved in other sectors due to the proximity of the urethra and rectum to the boost volume at mid-gland.

4. Data Export

Once the plan has been created, accepted and reviewed, the image set, structure set and plan should be exported in DICOM format along with the completed PAF using RTQA data transfer, see section 11.11

11.7.2. Brachyvision

Pre-trial QA instructions for planning the HDR brachytherapy arm of the PIVOTALboost trial using Brachyvision are as follows:

1. Import the study

Use the 'Import wizard' function in Brachyvision to import the planning CT and structure set. The image set has been delineated with the following structures:

Target volumes: Prostate, CTVp_1500, CTVpb_1900, GTVpb

OARs: Rectum, Urethra

The PTV has been created by adding a 3mm margin to the CTV, except posteriorly where a 0mm margin aims to limit dose to the anterior rectal wall. If this is consistent with your local protocol then continue planning with the structures provided. If you perform a different posterior margining process then please edit the CTVp_1500 accordingly, and **please comment on the margin used.**

2. Treatment planning

- This patient should be planned as a focal boost case (trial arm D2).
- Reconstruct all applicators ('insert', 'new applicator'), and use naming convention consistent with local protocol. Add dwell positions as appropriate.
- Optimise the dwell times as per local protocol, taking note of the planning guidelines below, and the prescription and dose constraints specified in the trial protocol (Version 1.1, 12th Sept. 2017, Pages 19/20).
- Complete the PIVOTALboost HDR plan assessment form, downloaded from the RTTQA web site.

3. Planning guidelines

The boost dose achieved will be limited by the OAR dose constraints and requirement to meet the whole gland coverage objectives.

When planning this case, the first priority is to meet the OAR dose constraints, the second priority is to achieve acceptable whole gland coverage, and the third priority is the boost dose.

Urethra D30 < 16.5 Gy can be considered a soft constraint, and higher D30 is allowed as long as the D10 < 17.5 Gy is achieved.

The PIVOTAL boost dose aims of CTVpb_1900 D90 ≥ 19 Gy, V19 Gy ≥ 90 % are not likely to be achieved for large volume boosts or boosts involving the anterior part of the gland due to urethral dose constraints.

4. Data transfer to QA team

Completed planning data (CT data set, RP file, RS file and RD file) and PAF should be exported from Brachyvision (‘file’, ‘export’, ‘patient’), and returned to the RTTQA team using RTQA data transfer, see section 11.11.

11.8. Patient Case Reviews

The outlining and planning for the first cohort of patients recruited by each trial centre will be subject to review by the RTQA team. This may be a prospective (i.e. pre-treatment) or timely retrospective review, to be advised by the RTQA team on a case-by-case basis. Patient specific QA measurements should also be provided if applicable.

To ensure a short response time for prospective reviews please notify the RTQA team when a patient has been identified, and please allow 2 weeks between submitting data and the RT treatment start date to allow time for amendments. Please send outlining for review in advance of RT treatment planning where possible. Failure to give the QA team sufficient notice of a case may result in delays in the case being reviewed. Should it not be possible to complete a review prior to the planned treatment start date, it is the PI’s responsibility to decide whether to start treatment as planned (prepared to re-plan for remaining treatment fractions if necessary) or to delay treatment start until review is complete.

For outlining reviews please send:

- Relevant screenshots of the diagnostic MRI study used to define the boost volume (if arm C2/D2) and a description of how these were used.*
- Planning MR images and DICOM registration object(s) (if used)
- Planning CT images
- DICOM structure set

* For patients randomised to the focal boost arms (C2, D2) only please send a separate PDF document with the **screenshots** of the diagnostic MRI images that were used for boost volume outlining, i.e. the T2W and the ADC map or, if not available, the diffusion (DWI) scan that was most helpful in outlining. It would be helpful if you could briefly explain what you wanted to do, e.g. size, position and superior / inferior extent of the boost volume(s). Please do not send the DICOM diagnostic MRI datasets.

For planning reviews please send:

- Planning CT images
- DICOM structure set
- DICOM dose matrix
- DICOM plan file
- Completed plan assessment form in Excel format as supplied

See Section 11.11 for data export instructions.

11.8.1. Planning/Outlining Review schedules:

Reviews will be performed as necessary to encompass all of the following scenarios:

IMRT review schedule:

A vs B centres
Retrospective review of the first pelvic node outlines and plan (B)

A vs B vs C2 vs D2
Prospective review of first 2 boost outlines (C2 or D2)
Retrospective review of the first prostate plan with boost (C2)
Retrospective review of first nodal outline (B or D2)
Retrospective review of the first pelvic plan with boost (D2)

HDR review schedule (in addition to the IMRT reviews above):

A vs B vs C1 vs D1
Retrospective review of the first HDR outline and plan (C1 or D1)
Retrospective review of the first pelvic node outlines and plan (B or D1)

A vs B vs C2 vs D2
Retrospective review of first 2 HDR boost outlines and plan (C2 or D2)
Retrospective review of the first pelvic node outlines and plan (B or D2)

Note the following:

1. All retrospective reviews are “timely”: data to be submitted within a week of treatment start and review to be done before another trial patient is treated.
2. Centres will be contacted after patient recruitment if a prospective review is needed.

11.9. Dosimetry Audit

All sites are required to have a recent dosimetry site visit by the RTTQA group. Sites which do not have this will be contacted individually by the RTTQA team to arrange an audit.

11.10. Ongoing Data Collection

Radiotherapy plan data will be collected (in DICOM format by electronic transfer) for all patients having radiotherapy within the trial. These data will be stored on a secure server by the sponsor. All patient data must be anonymised before transfer, and should be re-identified with the trial number.

Plans should ideally be submitted once they have been approved by the PI and had an independent check. Data associated with any re-plans during radiotherapy treatment should also be submitted.

Please send the following data:

- Planning MR images and DICOM registration object(s) (if used)
- Planning CT images
- DICOM structure set (ensure trial naming convention has been followed)
- DICOM dose matrix
- DICOM plan file

- Completed plan assessment form (original Excel format)

11.11. DICOM Data Export

Data transfer to the RTTQA group for the purpose of radiotherapy quality assurance for NIHR clinical trials is via a central secure transfer service. Anonymised and encrypted data passes through a firewall to a host server located in a secure NHS environment, to which access is restricted to authorised users. The transfer service is not a storage facility, it is a transit location; data will be deleted on download. It is the responsibility of the sender to anonymise and encrypt clinical trial data at source prior to upload. Encryption should be using 7zip/WinZip or equivalent.

All NHS radiotherapy centres have been given a centre-specific link and unique password to access the service.

Instructions for use:

1. Data must be anonymised at source and should be encrypted using 7zip/WinZip or equivalent
2. All files for a single patient must be zipped into one file
3. Files must be labelled with the Trial Name and Trial ID, e.g. PB02001
4. Follow unique centre link to the service

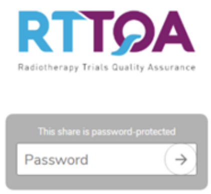

5. Insert unique centre password

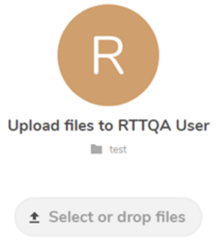

6. Upload files
7. Email RTTQA contact to confirm data uploaded and share password to unzip data

12. REFERENCES

ICRU (International Commission on Radiation Units and Measurements), Prescribing, recording and reporting photon beam therapy. Report No. 50, ICRU, Bethesda, MD, 1993.

ICRU (International Commission on Radiation Units and Measurements), Prescribing, recording and reporting photon beam therapy (supplement to ICRU Report 50). Report No. 62, ICRU, Bethesda, MD, 1999.

ICRU (International Commission on Radiation Units and Measurements), Prescribing, recording, and reporting photon-beam IMRT. Report No. 83, Journal of the ICRU Vol. 10, Oxford University Press, Oxford, U. K., 2010.

Mason J, Al-Qaisieh B, Bownes P, Wilson D, Buckley D L, Thwaites D, Carey B, and Henry A, Multi-parametric MRI- guided focal tumor boost using HDR prostate brachytherapy: a feasibility study. Brachytherapy, 2014. 13(2): p. 137-45.

Hoskin P J, Rojas A M, Bownes P J, Lowe G J, Ostler P J, and Bryant L, Randomised trial of external beam radiotherapy alone or combined with high-dose-rate brachytherapy boost for localised prostate cancer. Radiotherapy and Oncology : journal of the European Society for Therapeutic Radiology and Oncology, 2012. 103(2): p. 217-22.

13. APPENDIX A. (Diagnostic) MRI for PIVOTALboost

MRI minimum data set for Pivotalboost Study

Advances in technology (both in software and hardware) have led to the development of multiparametric MRI (mpMRI), which combines anatomical and functional assessment of the prostate including diffusion-weighted imaging (DWI) and dynamic contrast-enhanced (DCE) MRI sequences.

13.1. Patient Preparation

At present, there is no uniform consensus that covers every patient preparation issue.

To minimise motion artefact from bowel peristalsis, the use of an antispasmodic agent (e.g. Buscopan / Glucagon) can be beneficial in some patients. However, in many others it is not necessary.

The presence of faeces, fluid or gas in the rectum may induce artefactual distortion that can compromise the quality of the DWI images. Thus, some type of minimal preparation enema administered by the patient in the hours prior to the exam may be beneficial. However, an enema may also promote peristalsis, resulting in increased motion related artefacts in some instances. The patient should evacuate the rectum, if possible, just prior to the MRI exam.

Recommended:

- Administer Buscopan 20 mg im or Glucagon 1 mg im
- Ask patients to empty rectum if possible immediately prior to the MRI scan.

13.2. MRI sequences

Prostate MRI acquisition protocols may include T1W, T2W, DWI, and DCE sequences. At least one sequence should use a field-of-view (FOV) that permits evaluation of pelvic lymph nodes up to the level of the aortic bifurcation, e.g. to the level of L4/L5.

Recommended:

- T1W, T2W and DWI as a minimum.
- DCE is optional for Boost Volume definition purpose.

13.3. Magnetic Field Strength

Both 1.5T and 3T scanners can provide adequate images when acquisition parameters are optimized. 3T has advantages but is not a requirement for this Study.

Recommended:

- Either 1.5 T or 3 T MRI scanners are appropriate for this Study.

13.4. Endorectal Coil

When used in combination with surface phased array coils, endorectal coils increase Signal-to-Noise Ratio (SNR) in the prostate at any magnetic field strength. There may be diagnostic advantages in some patients. However, the endorectal coil causes considerable anatomical distortion and hence a surface coil is the preferred option. The use of endorectal coils for planning MRI image acquisition in this study is not permitted.

Recommended:

- Multichannel Surface phased array coils are adequate for MRI scanning in this Study.

13.5. Technical Specifications

T2W

Multiplanar (axial, coronal, and sagittal) images are generally acquired fast-spin-echo (FSE) or turbo-spin-echo (TSE). FSE or TSE provides high SNR and high spatial resolution images with significant T2 contrast. The imaging is performed using anywhere from 12 to 24 echoes with echo spacing around 8 ms to 12 ms. Typical repetition times range from 2 s to 4 s and effective echo times between 70 ms to 120 ms are used. Small FOVs of 12 cm to 16 cm, with in-plane matrices of 384 × 224 (frequency × phase) and slice thicknesses around 3 mm with no gap; in order to minimise blurring, excessive echo train lengths should be avoided. Use readout in the Anterior/Posterior direction to minimise motion artefacts (transaxial), and head/foot (sagittal).

Recommended: (Small FOV)

- Axial and at least one other orientation, sagittal preferred. Maximum 3 mm slice thickness. No gap
- Phase Resolution 100 %
- Sup-Inf coverage (specified by number of slices) and FOV should include all prostate + seminal vesicles (generally 12-16 cm)

T1W

Axial T1W images of the prostate may be obtained with or without fat suppression using spin echo or gradient echo sequences. Few (3 to 6) echo fast-spin echo (FSE) sequences are the preferred option, but classic spin-echo sequences, or 2D gradient echo sequences with large (> 50°) flip angles and TR periods (> 300 ms), are all acceptable. Coverage should include the pelvis and should extend up to include the pelvic rim; lower spatial resolution compared to T2W may be used to decrease acquisition time or increase anatomic coverage.

Recommended: (Large FOV)

- Axial images up to aortic bifurcation
- Maximum 6 mm slice thickness. 30% GAP
- FOV 340 mm to 400 mm, 512 image matrix with phase Resolution at least 60 %. Phase oversampling may be required.

DWI

The most common approach to DWI (Diffusion Weighted Imaging) of the prostate is the single-shot spin-echo EPI sequence. This sequence employs a water-selective excitation and refocusing pulse to generate a spin echo from a selected slice at echo times of the order of 60 ms to 100 ms, depending on the degree of diffusion weighting desired, known as the b-value.

Diffusion sensitization gradients are applied before and after the refocusing pulse and the latter half of the spin-echo is sampled with distinctly phase-encoded multiple-gradient echoes to generate a low

spatial resolution image. With DWI, each slice must be sampled with a baseline b=0 image and then sampled at one or several higher b-values, typically in the 500 to 1400 s/mm^2 range.

Combining high b-values images with the baseline image allows for calculation of the apparent diffusion coefficient (ADC) image, known also as the ADC map. This map is free of all T1, T2, and receiver coil sensitivities, and quantitative measurements of tissue water diffusion may be made from individual voxels, or regions-of-interest (ROIs), reported as the ROI's ADC in units of mm^2/s. More accurate ADC calculations and estimations of extrapolated high b-value images (≥ 1400 sec/mm2) may be used.

Recommended:

- Axial images. Locations should match or be similar to those used for T2W and DCE
- Minimum 3 b-values, reaching 700 s/mm^2 to 900 s/mm^2. They should be spread approximately equally within the range.
- Maximum slice thickness 5 mm. No gap
- In-plane resolution < 2 mm
- FOV: 16-22 cm, larger values are acceptable
- Produce an ADC map

DCE

DCE (Dynamic Contrast Enhancement) is optional; the role for boost volume delineation is limited. The acquisition of rapid T1W gradient echo scans before, during and after the intravenous administration of a low molecular weight gadolinium-based contrast agent). In order to detect early enhancing lesions in comparison to background prostatic tissue, temporal resolution should be < 10 seconds and preferably < 7 seconds per acquisition in order to depict focal early enhancement. Fat suppression and/or subtractions are recommended.

- TR/TE: < 100 ms/ < 5 ms
- Slice thickness: 3 mm. Locations should be the same as those used for DWI and T2W.
- Sup-Inf coverage and FOV: encompass the entire prostate gland and seminal vesicles
- In plane dimension: ≤ 2 mm X ≤2 mm
- Temporal resolution: ≤ 10 s (< 7 s is preferred)
- Total observation rate: > 2 min
- Dose: 0.1 mmol/kg standard contrast or equivalent
- Injection rate: 2-3 cm^3/s starting with continuous image data acquisition (should be the same for all exams).

14. APPENDIX B. GENERAL GUIDELINES FOR IGRT

14.1. Patients with Fiducials

14.1.1. Planar Imaging supplemented by Cone Beam CT (CBCT)

For all Trial Arms: Acquire a kV pair at every fraction and match online to the fiducial markers.

When marker matching, if any shift >1.0 cm a CBCT must be acquired and saved immediately after the fiducial shifts are applied. Treat patient, review offline before next fraction.

Note the following imaging and actions for specific Arms:

- Arms A, C1, and C2 post-HDR: image as above.
- Arms B, D1 and D2 post-HDR: Bone match and record values, THEN fiducial marker match and apply fiducial shifts. If >0.5 cm difference between bone and fiducial marker match, treat that fraction but annotate difference and use Section 0 to guide any further action. Repeat this process for up to 3 consecutive fractions subject to advice.
- Arm C2 with IMRT boost: as arm A, but after fiducial match is applied, acquire a CBCT to check CTVpb and rectum position (see below).
- Arm D2 with IMRT boost: as arm B, but after fiducial match is applied, acquire a CBCT to check CTVpb and rectum position (see below).

For IMRT boost arms C2 and D2 if rectum is encroaching on or overlaps with CTVpb, check for excess rectal gas/filling. Ask patient to get off bed if it looks like it is the filling that is causing the problem. Then repeat routine imaging for this Arm. If no improvement, treat and review offline and discuss with physics and clinician before next fraction. It may be necessary to monitor for up to 3 fractions at the discretion of the clinician. Clinician and/or Physics will advise if replan is required.

14.1.2. CBCT only

Patients with Fiducials

For all Trial Arms: Acquire a CBCT at every fraction and match online to the fiducial markers.

When marker matching, if any shift >1.0 cm, treat patient, review offline before next fraction.

Note the following imaging and actions for specific Arms:

- Arms A, C1, and C2 post-HDR: image as above.
- Arms B, D1, and D2 post-HDR: Match to bone and record values, THEN match on fiducials and apply fiducial shifts. If >0.5 cm difference between bone and fiducial match, treat that fraction but annotate difference and use section 0 to guide any

further action. Repeat this process for up to 3 consecutive fractions subject to advice.

- Arm C2 with IMRT boost: as arm A, but after fiducial match is applied, check CTVpb and rectum position (see below).
- Arm D2 with IMRT boost: as arm B, but after fiducial match is applied, check CTVpb and rectum position (see below).

For IMRT boost arms C2 and D2 if rectum is encroaching on or overlaps with CTVpb, check for excess rectal gas/filling. Ask patient to get off bed if it looks like it is the filling that is causing the problem. Then repeat routine imaging for this Arm. Treat and if no improvement alert expert immediately following treatment. They will review offline and discuss with clinician. It may be necessary to monitor for up to 3 fractions at the discretion of the clinician. Clinician and/or Physics will advise if replan is required.

14.2. Patients without Fiducials

14.2.1. CBCT

For patients without fiducials, CBCT matching

For all Trial Arms: Acquire a CBCT at every fraction and match online to the prostate. When matching, if any shift >1.0 cm, treat patient, review offline before next fraction.

Note the following imaging and actions for specific Arms:

- Arms A, C1, and C2 post-HDR: image as above.
- Arms B, D1, and D2 post-HDR: Match to bone and record values, THEN match to prostate and apply prostate shifts. If > 0.5 cm difference between bone and prostate match, treat that fraction but annotate difference and use section 0 to guide any further action. Repeat this process for up to 3 consecutive fractions subject to advice.
- Arm C2 with IMRT boost: as arm A, but after prostate match is applied, check CTVpb and rectum position (see below).
- Arm D2 with IMRT boost: as arm B, but after prostate match is applied, check CTVpb and rectum position (see below).

For IMRT boost arms C2 and D2 if rectum is encroaching on or overlaps with CTVpb, check for excess rectal gas/filling. Ask patient to get off bed if it looks like it is the filling that is causing the problem. Then repeat routine imaging for this Arm. Treat and if no improvement alert expert immediately following treatment. They will review offline and discuss with clinician. It may be necessary to monitor for up to 3 fractions at the discretion of the clinician. Clinician and/or Physics will advise if replan is required.

14.2.2. Ultrasound

For patients without fiducials, Ultrasound matching (Clarity)

For all Trial Arms: PLEASE SEND YOUR IMAGING PROTOCOL TO THE RTTQA PHYSICISTS

14.3. Proposed Actions to be taken during treatment with IGRT when the difference between the prostate match and the nodes match exceeds 5 mm

Since all PIVOTALboost patients in Arms B or D have disease in the prostate and are being treated prophylactically for the nodes, patients must be treated with the prostate matched. If the difference between the prostate and node match is within 5 mm, treatment continues without further action. If the difference exceeds this, there are various courses of action depending on the degree of shift. The recommended actions balance the risk of introducing unnecessary gaps in treatment (while images are being reviewed or a replan being done) with the risk of missing disease in the nodes.

5 mm < Difference < 10 mm	Apply shifts from prostate match, treat and refer for offline radiographer review. Monitor for 3 fractions and if difference is consistent perform a CBCT (if you normally use 2D kV imaging).
Difference > 10 mm	Set patient up again, repeat 2D match (if that is your normal practice) or CBCT (if that is your practice. If difference now less than 10 mm follow process above. If not, acquire CBCT, if not already done so. Do not treat and review offline to establish cause.

.

Offline radiographer review:

- Check the matched planning CT and CBCT.
- Do vessels within the CTVn lie outside PTVn by more than 1 mm over at least 3 slices?
- If so, try to determine cause of difference.
- Did the patient tense their buttocks at planning CT and now relaxed?
- Is rectal and/or bladder filling significantly different?

ACTION

- Instruct patient as appropriate (e.g. if bladder or rectal filling), aiming to reduce shift
- After 3 fractions decide whether shift is random or systematic or whether more daily CBCTs need to be taken and reviewed in order to help decision
- Refer for Clinical and Dosimetric review if out of tolerance shift persists after 3 fractions

Offline Clinical and Dosimetric review:

- Check the matched planning CT and CBCT.
- Do high risk significant portions of the CTVn on CBCT lie outside PTVn or, more specifically, outside the 95% (44.65 Gy) isodose?
- Is there a clinical reason? Does clinician need to see patient?

ACTION

- If differences are persistently >5 mm, do a repeat planning CT.
- Recalculate the current plan on the new CT and look again as to whether significant CTVn lies outside the original PTVn

- If shifts are random and significant portions of CTVn lie outside the original PTVn, consider replan with extended PTVn margins
- If shifts are systematic over at least 3 fractions, consider replan for new position
- Do not stop treatment while doing replan unless shifts are >10 mm on 3 repeated fractions.
